# Novel pyrazolone–thiophene Schiff base functionalized Fe_3_O_4_ nanocomposite: core–shell structure and multi-technique characterization

**DOI:** 10.1039/d5ra07567j

**Published:** 2025-11-07

**Authors:** Marwa Abdel-Motaal, Lotfi Beji, Noura Kouki, Medhat Asem, Mohammed E. Abdelmageed

**Affiliations:** a Department of Chemistry, College of Science, Qassim University Buraidah 51452 Saudi Arabia; b Department of Physics, College of Science, Qassim University Buraidah 51452 Saudi Arabia L.Beji@qu.edu.sa; c Department of Civil Engineering, College of Engineering and Information Technology, Onaizah Colleges Qassim 56447 Saudi Arabia; d Loyola Medicine, Loyola University Illinois USA; e Hot Laboratory Center, Atomic Energy Authority Cairo Egypt

## Abstract

This work reports the synthesis and structural characterization of a new magnetic nanocomposite, Fe_3_O_4_@PyTh, created by forming a surface-functionalized magnetic nanocomposite with a pyrazolone–thiophene Schiff base (PyTh). The ligand was produced *via* a one-pot condensation reaction and attached as a shell on the Fe_3_O_4_ core, forming an organic–inorganic hybrid interface. Comprehensive characterization using Nuclear Magnetic Resonance (NMR), Fourier-Transform Infrared Spectroscopy (FTIR), X-Ray Diffraction (XRD), Scanning Electron Microscopy (SEM), Energy-Dispersive X-ray Spectroscopy (EDX), Transmission Electron Microscopy (TEM), and Selected Area Electron Diffraction (SAED) confirmed successful ligand attachment, the preservation of the cubic spinel Fe_3_O_4_ core, and the formation of a uniform organic shell. TEM analysis showed an increase in size from 7.5 to 9.1 nm after PyTh modification, while EDX mapping confirmed the presence of nitrogen, sulfur, and carbon, indicating effective functionalization. This study highlights the structural stability and hybrid nature of the Fe_3_O_4_@PyTh surface-functionalized magnetic nanocomposite, offering experimental structural insights into Schiff base functionalization as an adaptable route for designing advanced magnetic hybrid materials.

## Abstract 2

This work reports the synthesis and structural characterization of a new magnetic nanocomposite, Fe_3_O_4_@PyTh, created by forming a surface-functionalized magnetic nanocomposite with a pyrazolone–thiophene Schiff base (PyTh).

## Graphical abstract

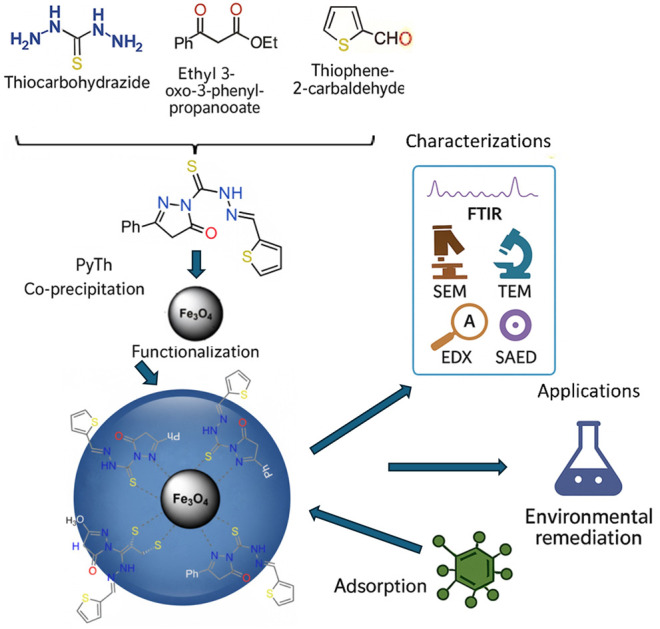

## 1 Introduction

The ongoing pollution of global water bodies by harmful organic compounds has become a significant environmental and public health concern, increasing the demand for advanced materials for treatment. Pollutants such as synthetic dyes, pharmaceutical residues, pesticides, and phenolic derivatives are often released by industries like textiles, petrochemicals, and pharmaceuticals, disrupting ecosystems and posing serious risks to both human health and biodiversity. These contaminants are known for their toxicity, often being carcinogenic, and they are highly persistent in the environment, highlighting the urgent need for effective and sustainable treatment methods.^1,2^ In this context, designing functional magnetic nanomaterials is a promising research path. This study focuses on synthesizing and fundamentally characterizing such material, identifying its structural properties as a foundation for future application-driven research.

Meanwhile, nanotechnology has created new possibilities in materials design, where nanoparticles with adjustable sizes, shapes, and surfaces are engineered for improved stability, reactivity, and functional interfaces.^3–7^ Among these materials, magnetic iron oxide nanoparticles—especially magnetite (Fe_3_O_4_)—have gained significant attention because of their superparamagnetic behavior, excellent chemical stability, and ease of recovery from water with external magnetic fields.^8–10^ In practice, Fe_3_O_4_-based nanosystems have shown high efficiency across various application modes, including the adsorptive removal of persistent organic pollutants and dyes, catalytic and advanced oxidation pathways, and magnetic separations that allow for rapid recovery and reuse, as documented in recent reports.^11–15^ Bare Fe_3_O_4_ nanoparticles are limited by their mostly unreactive surface chemistry, which reduces their ability to interact with many organic contaminants. To improve this, surface functionalization with organic ligands has been shown to increase adsorption capacity and expand potential applications.^16–18^ Notably, donor-rich Schiff bases are effective in enhancing interfacial reactivity through coordination with surface Fe^3+^ centers. Recent studies further demonstrate Schiff-base/MNP hybrids used for adsorption and catalysis—for example, Schiff-base magnetic nanocatalysts,^19^ triazole–Schiff-base–Pd on Fe_3_O_4_,^20^ chitosan–Schiff-base magnetic biosorbents,^21^ g-C_3_N_4_/Fe_3_O_4_–Schiff-base composites,^22^ and Pd@dppe@Fe_3_O_4_ (ref. 23)—highlighting the significance of ligand-engineered magnetic platforms for separable remediation and catalysis.

Schiff bases have become versatile and effective agents for functionalization. Their structures typically include multiple coordination sites—such as imine (–CH=N–), carbonyl (C=O), and heterocyclic groups—that enable strong interactions with various pollutants through mechanisms like hydrogen bonding, π–π stacking, and electrostatic forces.^6^ Pyrazolone-based Schiff bases offer additional advantages due to their rich π-conjugated systems and sulfur-containing groups, which enhance their affinity for aromatic and heterocyclic pollutants.^24,25^ Incorporating thiophene units introduces extra electron-rich aromaticity,^26,27^ expanding the chemical versatility of these ligands for nanoparticle functionalization. In this study, we present a new nanocomposite: magnetite (Fe_3_O_4_) nanoparticles functionalized with a pyrazolone–thiophene Schiff base (PyTh), forming a core–shell hybrid material called Fe_3_O_4_@PyTh. The resulting material is best described as a surface-functionalized Fe_3_O_4_-based nanocomposite, with the PyTh ligand forming a thin organic shell around the magnetic core. This design combines the magnetic core of Fe_3_O_4_, allowing for easy recovery and reuse, with the PyTh ligand, which provides a chemically active surface rich in donor atoms and aromatic sites. Although many Fe_3_O_4_-based composites—functionalized with agents such as amino-silanes, chitosan derivatives, and traditional Schiff bases—have been extensively studied, the specific use of a pyrazolone–thiophene Schiff base for nanoparticle functionalization remains largely unexplored, highlighting the novelty and potential importance of this work.^28–32^ Extensive DFT studies have characterized Fe_3_O_4_ surface terminations and molecular adsorption on (001)/(111) facets;^33–36^ our upcoming computational work builds on this foundation to model PyTh binding geometries and benchmark them against a non-thiophene analog to quantify the thiophene contribution. In this work, we synthesize a pyrazolone–thiophene (PyTh) Schiff base and immobilize it onto Fe_3_O_4_ to form a core–shell Fe_3_O_4_@PyTh nanocomposite. Unlike recent Schiff-base/MNP systems,^19–23^ the PyTh shell offers a π-conjugated S,N-donor interface, and our study uses quantitative SAED/XRD analysis supported by custom Python scripts to assess interfacial strain and confirm spinel preservation—emphasizing structural rather than application-focused novelty.

The Fe_3_O_4_@PyTh nanocomposite was prepared through a simple multi-step process: first, the pyrazolone–thiophene Schiff base was synthesized *via* a one-pot condensation reaction involving thiocarbohydrazide, ethyl 3-oxo-3-phenylpropanoate, and thiophene-2-carbaldehyde; next, magnetite nanoparticles were produced by co-precipitation; and finally, the PyTh ligand was attached to the surface of Fe_3_O_4_. Co-precipitation was chosen because it is quick and simple to operate, provides high space–time yield at moderate temperatures, is energy-efficient, and avoids toxic reagents or byproducts; importantly, it ensures batch-to-batch consistency and easily produces surfaces suitable for subsequent ligand functionalization—criteria central to this work. These practical benefits align with recent evaluations of convenient wet-chemical routes for nanomaterial deposition.^37^

The resulting composite was thoroughly characterized using various techniques, including FTIR, X-ray diffraction (XRD), scanning electron microscopy (SEM), transmission electron microscopy (TEM), energy-dispersive X-ray spectroscopy (EDX), nuclear magnetic resonance (NMR), and selected area electron diffraction (SAED), confirming its structural stability and successful functionalization.

To expand the use of Schiff base-functionalized nanomaterials in catalysis, sensing, and related nanotechnology fields,^7,9^ this work focuses solely on the synthesis, functionalization, and detailed structural analysis of Fe_3_O_4_@PyTh. This marks a major shift from our previous work on biological applications of pyrazolone derivatives,^26,27,31^ moving toward the development of functional nanomaterials for environmental technology. The main goal is to definitively confirm the creation of this new hybrid material and to clarify its core–shell structure, crystallinity, and surface chemistry. This foundational study is a critical first step, and future research will measure its performance in specific applications—such as the adsorption of organic contaminants—where the functionalized surface is expected to offer significant benefits over bare Fe_3_O_4_ nanoparticles.

## 2 Materials and methods

### 2.1 Chemicals and reagents

All chemicals and reagents were purchased from Sigma-Aldrich and used without further purification. Thin-layer chromatography (TLC) plates, pre-coated with silica gel (SiO_2_) on aluminum sheets, were also obtained from Sigma-Aldrich. TLC spots were visualized under ultraviolet (UV) light. Melting points were determined using a Stuart SMP30 digital melting point apparatus with open glass capillaries.^38^

### 2.2 Instrumentation

#### 2.2.1 Mass spectrometry (MS)

Electrospray ionization mass spectra (ESI-MS) were obtained using a Varian MAT 311 mass spectrometer operating at 70 eV, located at the Microanalytical Center, Faculty of Science, Cairo University. Samples were dissolved in HPLC-grade methanol and directly infused at a flow rate of 5 μL min^−1^. The capillary temperature was set to 250 °C, and the spray voltage was 4.5 kV.

#### 2.2.2 Nuclear magnetic resonance (NMR) spectroscopy

^1^H and ^13^C NMR spectra were recorded at room temperature using a JNM-ECA500II (Mansoura University NMR unit) spectrometer (500 MHz for ^1^H, 125 MHz for ^13^C) with DMSO-d_6_ as the solvent. Tetramethylsilane (TMS) served as the internal standard, and chemical shifts are reported in *δ* (ppm).^39^ The spectra were acquired with a 30° pulse angle, a relaxation delay of 2 seconds, and 64 scans for ^1^H and 1024 scans for ^13^C NMR.

#### 2.2.3 X-ray diffraction (XRD)

XRD patterns were collected using a Rigaku diffractometer with Cu-Kα radiation (*λ* = 1.5406 Å). Data were recorded in the 2*θ* range of 10° to 90° with a step size of 0.02° and a scanning speed of 2° min^−1^. Powder samples were lightly pressed onto a zero-background silicon sample holder.

#### 2.2.4 Scanning electron microscopy (SEM)

SEM images were taken using a JEOL JSM-6390 scanning electron microscope. Samples were prepared by dispersing the powder in ethanol, depositing a drop onto an aluminum stub, and allowing it to dry in air. The samples were then sputter-coated with a thin layer of gold to enhance conductivity. Imaging was performed at an accelerating voltage of 15 kV.

#### 2.2.5 Fourier-transform infrared spectroscopy (FTIR)

FTIR spectra were recorded in transmission mode over the range of 400–4000 cm^−1^ using a BRUKER EQUINOX-55 spectrometer. Samples were prepared by mixing the powder with dry KBr (approximately 1 : 100 sample-to-KBr ratio) and compressing the mixture into a pellet. Each spectrum was the result of 32 scans at a resolution of 4 cm^−1^ and is reported in % transmittance.

#### 2.2.6 Transmission electron microscopy (TEM)

TEM analysis was conducted with a JEOL JEM-1010 transmission electron microscope to examine particle morphology. Samples were prepared by dispersing the powder in ethanol using ultrasonication for 10 minutes. A drop of the suspension was then placed onto a carbon-coated copper grid and allowed to dry at room temperature. Imaging was carried out at an accelerating voltage of 100 kV.

### 2.3 Synthesis of the Schiff base (PyTh)

The pyrazolone–thiophene Schiff base (PyTh), namely 5-oxo-3-phenyl-*N*′-(thiophen-2-ylmethylene)-4,5-dihydro-1*H*-pyrazole-1-carbothiohydrazide, was synthesized through a two-step process.

#### 2.3.1 Step 1: synthesis of the pyrazolone intermediate

A mixture of ethyl benzoyl acetate (0.01 mol) and thiocarbohydrazide (0.01 mol) was dissolved in 30 mL of absolute ethanol, followed by the addition of 1 mL of concentrated HCl. The solution was then refluxed at 85 °C for 1 hour, with the reaction progress monitored by TLC.

#### 2.3.2 Step 2: formation of PyTh

To the reaction mixture, 2-formyl thiophene (0.01 mol) was added without isolating the intermediate. Reflux was then continued for an additional 30 minutes, monitored by TLC. After cooling, the dark yellow precipitate was collected by filtration and recrystallized from ethanol to produce pure PyTh with an 85% yield (melting point: 228–230 °C). Ethanol was selected as a green and recoverable solvent for this condensation, achieving an atom economy of approximately 95% (water being the only by-product). Although water offers an even greener alternative, it was unsuitable for this synthesis due to the limited solubility of the reactants and the susceptibility of the imine bond to hydrolysis during aqueous workup. Beyond TLC and a sharp melting range, purity/identity were confirmed by clean ^1^H/^13^C NMR spectra without extraneous resonances and by ESI-MS consistent with the assigned formula; HPLC was not performed in this study. In this work, no further solvent or catalyst screening was undertaken; the one-pot ethanol/HCl protocol was selected for its operational simplicity and reproducibly high isolated yield.

### 2.4 Synthesis of magnetite (Fe_3_O_4_) nanoparticles

Fe_3_O_4_ nanoparticles were synthesized using a co-precipitation method as described in previous studies:^28,31^

#### 2.4.1 Preparation of iron solutions

Iron(iii) chloride hexahydrate (FeCl_3_·6H_2_O, 8 mmol; 2.16 g) and iron(ii) chloride tetrahydrate (FeCl_2_·4H_2_O, 4 mmol; 0.79 g) were each dissolved in small amounts of deionized water.

#### 2.4.2 Co-precipitation

A 2 M NaOH solution was gradually added to the mixed iron salt solution while stirring vigorously, adjusting the pH to a range of 8 to 14. This alkaline window is used to guarantee complete Fe^2+^/Fe^3+^ conversion to Fe_3_O_4_ and to suppress parasitic ferric (oxy)hydroxide formation during nucleation and aging; it reflects the commonly reported stability domain for magnetite in co-precipitation syntheses.^28,29^ A black precipitate of Fe_3_O_4_ appeared immediately.

#### 2.4.3 Colloidal stability and pH

Because the surface charge (*ζ*-potential) of Fe_3_O_4_ is highly pH-dependent—approaching zero near neutral pH but becoming more negative under alkaline conditions—the chosen basic environment encourages electrostatic stabilization and prevents pre-functionalization aggregation. Combined with the use of deoxygenated water and immediate washing, these conditions help reduce both oxidation and particle aggregation before PyTh anchoring. Although dynamic light scattering (DLS) and *ζ*-potential measurements were not performed in this work, this limitation is recognized, and such analyses are planned for a future application study.

#### 2.4.4 Purification

The precipitate was separated with a magnet, thoroughly rinsed with deoxygenated distilled water until a neutral pH was reached, then rinsed with acetone. The final product was dried at 60–70 °C in a drying oven.

### 2.5 Synthesis of Fe_3_O_4_@PyTh nanocomposite

The Fe_3_O_4_@PyTh nanocomposite was created by surface functionalization of the synthesized Fe_3_O_4_ nanoparticles as follows:

#### 2.5.1 Dispersion of Fe_3_O_4_

Two grams of Fe_3_O_4_ nanoparticles were dispersed in 50 mL of ethanol and stirred thoroughly for 2 hours.

#### 2.5.2 Functionalization with PyTh

In a separate flask, 8 mmol of PyTh was dissolved in 15 mL of hot absolute ethanol. This solution was then added dropwise to the Fe_3_O_4_ suspension, and the mixture was refluxed in ethanol (∼78–80 °C) in a water bath for 5 hours to facilitate surface immobilization of the Schiff base. Ethanol provides consistent dispersion and stable anchoring conditions; in initial trials, only aqueous media caused quick aggregation and lowered ligand retention. No time-dependent FTIR kinetic series were acquired; 5 h was adopted as a fixed, literature-consistent duration to ensure complete surface attachment while avoiding prolonged heating of the organic shell.

#### 2.5.3 Separation and purification

The Fe_3_O_4_@PyTh nanocomposite was easily separated from the solution using an external magnet, confirming that its magnetic properties were retained after functionalization. It was then washed repeatedly with water and ethanol and dried at 80 °C.

The mass ratio of Fe_3_O_4_ to PyTh used in the synthesis was 1 : 0.45 (2 g : 0.9 g). This ratio was selected based on preliminary optimization to promote a high level of surface coverage while minimizing the formation of physisorbed multilayers.

Thermogravimetric analysis (TGA) and differential scanning calorimetry (DSC) were not used in this study. Future research will employ these techniques—including derivative TGA and combined DSC-TGA under N_2_ and air—to quantify the PyTh content and thoroughly map the thermal transitions and stability range of the Fe_3_O_4_@PyTh hybrid.

Future process intensification will compare ethanol–water mixtures, bio-derived solvents (*e.g.*, 2-MeTHF or deep-eutectic media), and solvent recovery mass balances (PMI/E-factor) to assess scalable, water-efficient options.

### 2.6 Synthetic route and characterization summary

The stepwise synthesis procedure follows established methods for Schiff base-functionalized nanomaterials.^24,40^ Ethyl benzoyl acetate was reacted with thiocarbohydrazide in the presence of concentrated HCl, then 2-formyl thiophene was added *in situ*, forming the expected intermediate (A).

The reaction progress was monitored by thin-layer chromatography (TLC) to confirm completion. The resulting Schiff base was isolated, and its structure was verified using infrared (IR) spectroscopy, nuclear magnetic resonance (NMR) spectroscopy, and mass spectrometry (MS). Characteristic absorption peaks for C=O, N–H, CH_2_, CH=N, and aromatic protons provided clear evidence of successful PyTh formation.

Magnetite (Fe_3_O_4_) nanoparticles were synthesized *via* co-precipitation of ferric and ferrous ions in alkaline conditions. The nanoparticles were magnetically separated, washed, and then functionalized with the PyTh ligand by refluxing in ethanol, forming the Fe_3_O_4_@PyTh nanocomposite. Successful surface modification was confirmed by FTIR analysis, which showed significant shifts in vibrational bands, indicating effective bonding of PyTh onto the Fe_3_O_4_ surface.

**Scheme 1:**
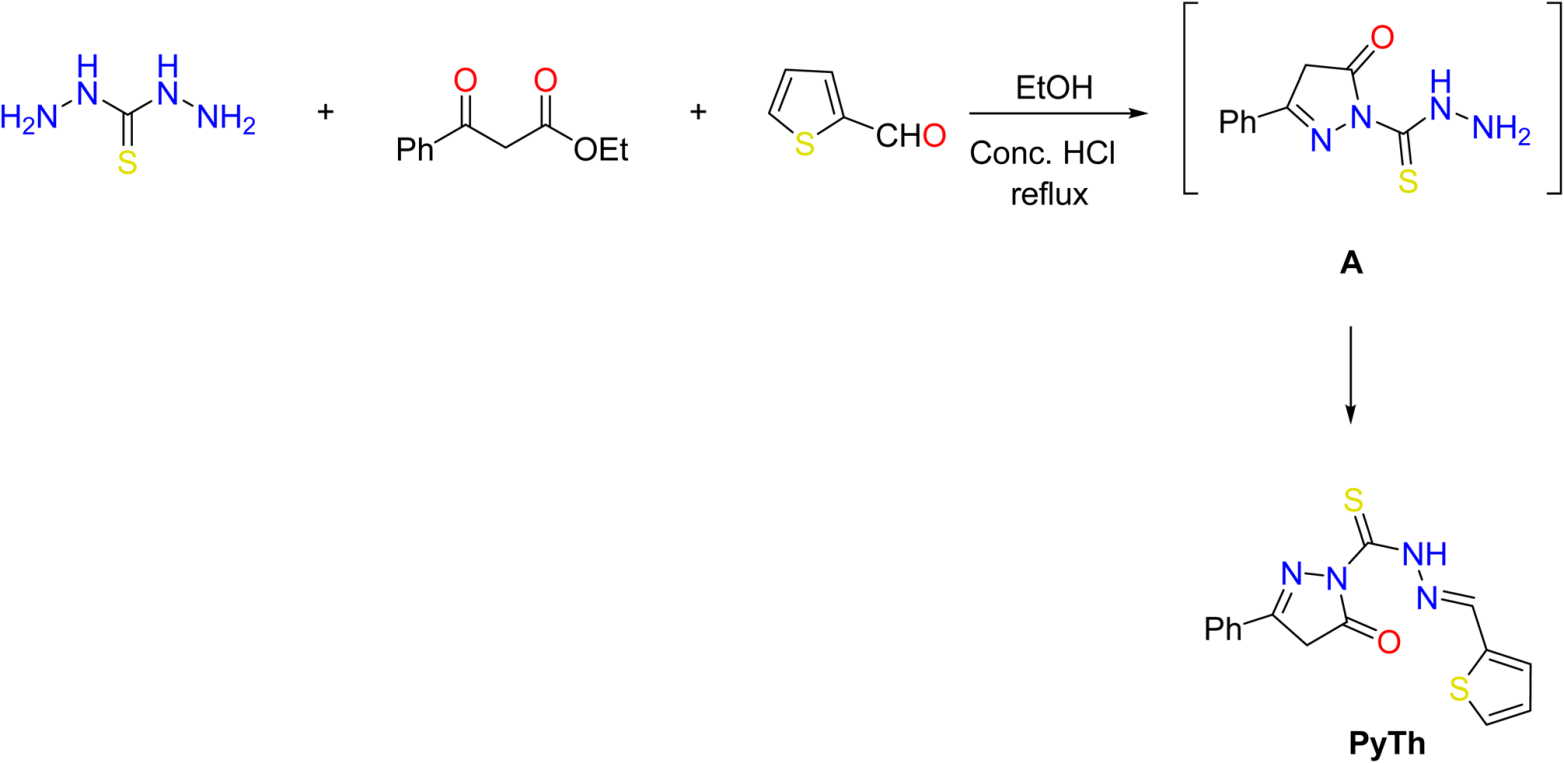
Synthesis of the pyrazolone Schiff base adsorbent PhTy.

**Scheme 2:**
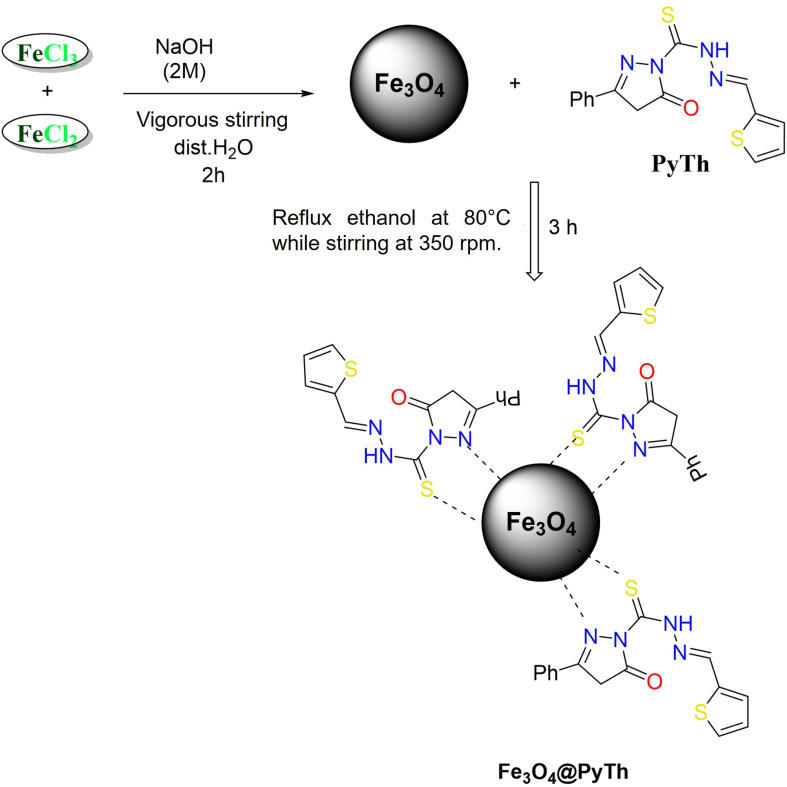
Anchoring of the pyrazolone Schiff base (PyTh) on the surface of Fe_3_O_4_ magnetic nanoparticles.

The complete synthetic route for the Fe_3_O_4_@PyTh nanocomposite is outlined in Schemes 1 and 2. In brief, the procedure involved three main steps: (i) the one-pot synthesis of the pyrazolone–thiophene Schiff base through condensation of thiocarbohydrazide, ethyl 3-oxo-3-phenylpropanoate, and thiophene-2-carbaldehyde; (ii) preparing Fe_3_O_4_ nanoparticles *via* co-precipitation; and (iii) functionalizing the Fe_3_O_4_ surface with PyTh to produce the final Fe_3_O_4_@PyTh nanomaterial. Scheme 1 shows the synthetic pathway for the pyrazolone–thiophene Schiff base (PyTh), starting from thiocarbohydrazide, ethyl 3-oxo-3-phenylpropanoate, and thiophene-2-carbaldehyde. The synthesis occurs *via* a one-pot condensation in ethanol, catalyzed by hydrochloric acid, leading to the PyTh Schiff base. This process introduces key functional groups—including N–H, C=O, C=N (Schiff base linkage), and sulfur (S)—which are essential for subsequent coordination with metal oxide surfaces like Fe_3_O_4_. These functional groups provide multiple active binding sites, improving the material's potential for strong molecular interactions, especially in adsorption applications. Importantly, the C=N bond supports π–π stacking interactions with aromatic systems, a crucial mechanism for capturing organic contaminants. The resulting PyTh structure is chemically stable and suitable for anchoring onto Fe_3_O_4_ nanoparticles, forming the basis for the development of advanced hybrid materials.

Scheme 2 illustrates the functionalization of Fe_3_O_4_ nanoparticles with the synthesized PyTh Schiff base. The Fe_3_O_4_ nanoparticles, produced through co-precipitation of FeCl_3_ and FeCl_2_ under alkaline conditions, are dispersed in ethanol, after which the PyTh ligand is added. Attachment of PyTh onto the nanoparticle surfaces is believed to occur *via* coordination between the Fe_3_O_4_ surface sites and the functional groups of the Schiff base, such as N–H and C=O. This surface modification yields a composite material, Fe_3_O_4_@PyTh, that combines the magnetic properties of Fe_3_O_4_ with the adsorption capabilities of PyTh.

The functionalization process successfully transforms bare Fe_3_O_4_ nanoparticles into a versatile nano-adsorbent. The magnetic core allows for easy recovery from water solutions *via* external magnetic fields, making Fe_3_O_4_@PyTh both effective and reusable for water treatment. Schemes 1 and 2 demonstrate a step-by-step method for synthesizing and functionalizing nanomaterials, emphasizing a promising approach for environmental cleanup. This technique combines the benefits of chemical functionalization and magnetic separation, offering a practical and innovative solution for advanced wastewater treatment.

## 3 Results and discussion

The successful synthesis of the pyrazolone–thiophene Schiff base (PyTh) and its incorporation with magnetite nanoparticles to create the Fe_3_O_4_@PyTh nanocomposite were confirmed through various structural and morphological analyses. This section begins with detailed molecular characterization of PyTh using NMR, ESI-MS, and FTIR spectroscopy, which verify the presence of essential functional groups necessary for its role as a surface ligand. Next, the structural and surface features of the synthesized Fe_3_O_4_ nanoparticles and the Fe_3_O_4_@PyTh nanocomposite are examined. Comparative FTIR analysis demonstrates the successful functionalization of the nanoparticle surface, while XRD, SEM, EDX, and TEM provide further insights into the crystallinity, morphology, elemental composition, and nanoscale characteristics of the final material.^32,40^

### 3.1 NMR, ESI-MS and FTIR of the Schiff base (PyTh)

The pyrazolone–thiophene Schiff base (PyTh) was synthesized by condensing thiocarbohydrazide with ethyl 3-oxo-3-phenylpropanoate in refluxing ethanol, using concentrated hydrochloric acid as a catalyst. Without isolating the intermediate (A), 2-formylthiophene was added *in situ* to complete the reaction. The structure of the resulting Schiff base was confirmed through infrared (IR) spectroscopy, electrospray ionization mass spectrometry (ESI-MS), and nuclear magnetic resonance (NMR) spectroscopy.

#### 3.1.1 ^1^H NMR spectrum interpretation (500 MHz, DMSO-d_6_)

The structural confirmation of the synthesized pyrazolone–thiophene Schiff base (PyTh) was further supported by ^1^H nuclear magnetic resonance (^1^H NMR) spectroscopy, recorded at 500 MHz using DMSO-d_6_ as the solvent. The spectrum provides detailed insights into the proton environments of the molecule, allowing for precise assignment of resonances to specific structural motifs.

**Fig. 1:**
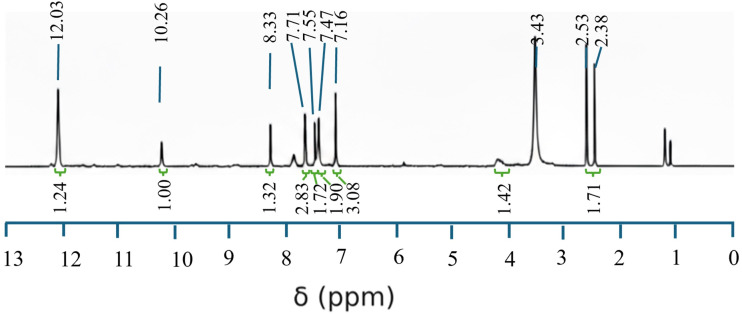
^1^H NMR spectrum of the pyrazolone–thiophene Schiff base (PyTh), illustrating characteristic resonances for aromatic, aliphatic, and functional group protons.

The ^1^H NMR spectrum of PyTh (Fig. 1) clearly confirms the proposed molecular structure, displaying distinct signals for different proton environments. The peaks at *δ* 2.53 ppm and *δ* 3.43 ppm correspond to residual solvent (DMSO-d_5_) and water (H_2_O), respectively, and are not part of the molecule. A small triplet observed around *δ* 1.2 ppm is attributed to trace residual solvent impurities, likely diethyl ether or ethanol, from the synthesis. These signals are not part of the final molecular structure.

• *δ* 2.38 (s, 3H, –CH_3_): the singlet at *δ* 2.38 ppm, integrating to about three protons, is assigned to the methyl (–CH_3_) group on the pyrazolone ring. The chemical shift is typical of a methyl group attached to an sp^2^ carbon within a heterocyclic system.

• *δ* 7.16–7.71 (m, ∼9H, Ar–H): the series of overlapping multiplets between *δ* 7.16 and 7.71 ppm corresponds to the protons of the phenyl and thiophene aromatic rings. The integration for this region is approximately nine protons, representing the five protons from the monosubstituted phenyl ring and the expected protons from the thiophene ring system.

• *δ* 8.33 (s, 1H, CH=N): the sharp singlet at *δ* 8.33 ppm, with an integration indicating one proton, is clearly assigned to the imine proton (CH=N) of the Schiff base linkage. Its downfield position is typical of an azomethine proton and aligns with values reported for similar Schiff base structures.^38,41^

• *δ* 10.26 (s, 1H, NH) and *δ* 12.03 (s, 1H, SH): the spectrum shows two distinct singlets in the downfield region, each corresponding to one proton. The signal at *δ* 10.26 ppm is attributed to the amide proton (N–H) of the pyrazolone ring. The signal at *δ* 12.03 ppm is attributed to the thiol proton (S–H). The presence of these two signals, along with the absence of an enolic OH signal (usually around 14–15 ppm in such systems), strongly suggests that the PyTh ligand mainly exists in the keto–enol/thiol tautomeric form in DMSO solution.^25,31^

The spectral data strongly support the proposed molecular structure of PyTh and confirm the successful formation of the Schiff base. The ^1^H NMR spectrum provides clear evidence for the ligand's tautomeric state. The presence of two distinct downfield singlets—an amide proton (N–H) at *δ* 10.26 ppm and a thiol proton (S–H) at *δ* 12.03 ppm—along with the notable absence of a characteristic enolic OH signal, indicates that PyTh mainly exists in the keto–enol/thiol tautomeric form in solution. This interpretation aligns with reports on structurally similar heterocyclic systems.^25,31^ The assignment is further supported by the FTIR data, where a strong C=O stretching vibration is observed near 1670 cm^−1^, confirming the dominance of the keto form.

#### 3.1.2 ^13^C NMR spectrum interpretation (125 MHz, DMSO-d_6_)

To further confirm the molecular structure of the synthesized pyrazolone–thiophene Schiff base (PyTh), ^13^C nuclear magnetic resonance (^13^C NMR) spectroscopy was performed at 125 MHz in DMSO-d_6_. The spectrum provides detailed insight into the carbon environments within the molecule, confirming the presence of aromatic, aliphatic, and functional group carbons as expected from the proposed structure. Key resonances were assigned based on chemical shifts and linked to specific carbon atoms in the PyTh framework.

**Fig. 2:**
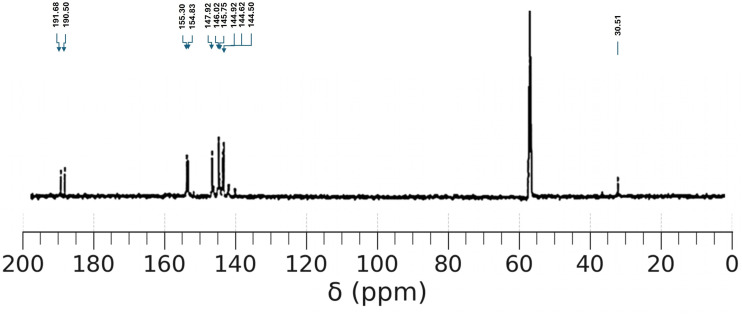
^13^C NMR spectrum of the pyrazolone–thiophene Schiff base (PyTh), displaying characteristic carbon resonances that confirm the molecular architecture.

The ^13^C NMR spectrum of PyTh (Fig. 2) shows distinct signals that correspond to the expected carbon skeleton of the Schiff base. The large signal at approximately 55–60 ppm is attributed to the deuterated solvent (likely methanol-d_4_) and is not part of the molecule.

• *δ* 30.51: this upfield signal corresponds to the methyl (–CH_3_) group on the pyrazolone ring. Its chemical shift is characteristic of an aliphatic carbon attached to an sp^2^-hybridized system.

• *δ* 144.50–147.92: this cluster of peaks in the aromatic region represents the carbon atoms of the phenyl ring and the thiophene ring. The complexity and overlap suggest multiple distinct carbon environments, which is expected for these two different aromatic systems.

• *δ* 154.83 and 155.30: these two downfield signals are characteristic of quaternary carbons in electron-deficient environments. The peak at *δ* 155.30 is assigned to the imine carbon (C=N) of the Schiff base linkage. The adjacent peak at *δ* 154.83 likely corresponds to the quaternary carbon of the pyrazolone ring attached to the two nitrogen atoms.

• *δ* 190.50 and 191.68: these signals in the far downfield region are unequivocally assigned to carbonyl (C=O) and thiocarbonyl (C=S) carbons. The resonance at *δ* 191.68 is assigned to the C=O group of the pyrazolone ring. The signal at *δ* 190.50 is attributed to the C=S (thione) carbon. The presence of both signals confirms the keto–thione tautomeric form of the ligand, which is consistent with the ^1^H NMR data.

##### A Structural interpretation

• Imine linkage (C=N): the sharp singlet at *δ* 8.33 ppm in the ^1^H NMR spectrum, along with a distinct carbon signal at *δ* 155.30 ppm in the ^13^C NMR, definitively confirms the formation of the imine (Schiff base) linkage.

• Thiophene and phenyl rings: the overlapping complex multiplets between *δ* 7.16 and 7.71 ppm in the ^1^H NMR spectrum, integrating to about nine protons, represent the aromatic protons of both the thiophene and the monosubstituted phenyl rings. This is further supported by the group of aromatic signals in the ^13^C NMR spectrum between *δ* 144.5 and 147.9 ppm.

• Methyl group (–CH_3_): the singlet at *δ* 2.38 ppm in the ^1^H NMR, integrating to three protons, and the corresponding upfield signal at *δ* 30.51 ppm in the ^13^C NMR, confirm the presence of the methyl group on the pyrazolone ring.

• Keto–enol/thiol tautomerism: the ^1^H NMR spectrum displays two separate, exchangeable protons in the downfield region: an amide (N–H) proton at *δ* 10.26 ppm and a thiol (S–H) proton at *δ* 12.03 ppm. The presence of these signals, along with the absence of a typical enolic OH peak, strongly indicates that the ligand exists in a keto–enol/thiol tautomeric form.

• Carbonyl (C=O) and thione (C=S) groups: the presence of the keto–thione structure is clearly demonstrated by two distinct downfield signals in the ^13^C NMR spectrum. The peak at *δ* 191.68 ppm corresponds to the carbonyl (C=O) carbon, while the peak at *δ* 190.50 ppm corresponds to the thione (C=S) carbon.

##### B Structural hypothesis

Based on the combined spectral data, the structure of PyTh is confirmed. The molecule contains a Schiff base (C=N) linkage, evidenced by characteristic ^1^H and ^13^C NMR signals, resulting from the successful condensation of the amine and aldehyde precursors. The structure includes both a phenyl and a thiophene ring, confirmed by the aromatic proton and carbon signals. Importantly, the data demonstrate the molecule's specific tautomeric form. The presence of distinct amide (N–H) and thiol (S–H) protons in the ^1^H NMR spectrum, along with carbonyl (C=O) and thione (C=S) signals in the ^13^C NMR spectrum, confirms that PyTh exists in a stable keto–enol/thiol tautomeric form. This structure is stabilized by an extended π-conjugation system that covers the aromatic rings, the pyrazolone core, and the imine linkage, offering a strong framework for its intended functional applications.

#### 3.1.3 ESI-MS analysis of PyTh

Electrospray ionization mass spectrometry (ESI-MS) was used to confirm the molecular weight of the synthesized pyrazolone–thiophene Schiff base (PyTh). Alongside NMR analysis, ESI-MS acted as an orthogonal method to verify the compound's identity and evaluate sample purity.

The mass spectrum provides precise molecular ion data, verifying the expected molecular formula and offering insight into the compound's fragmentation pattern.

**Fig. 3:**
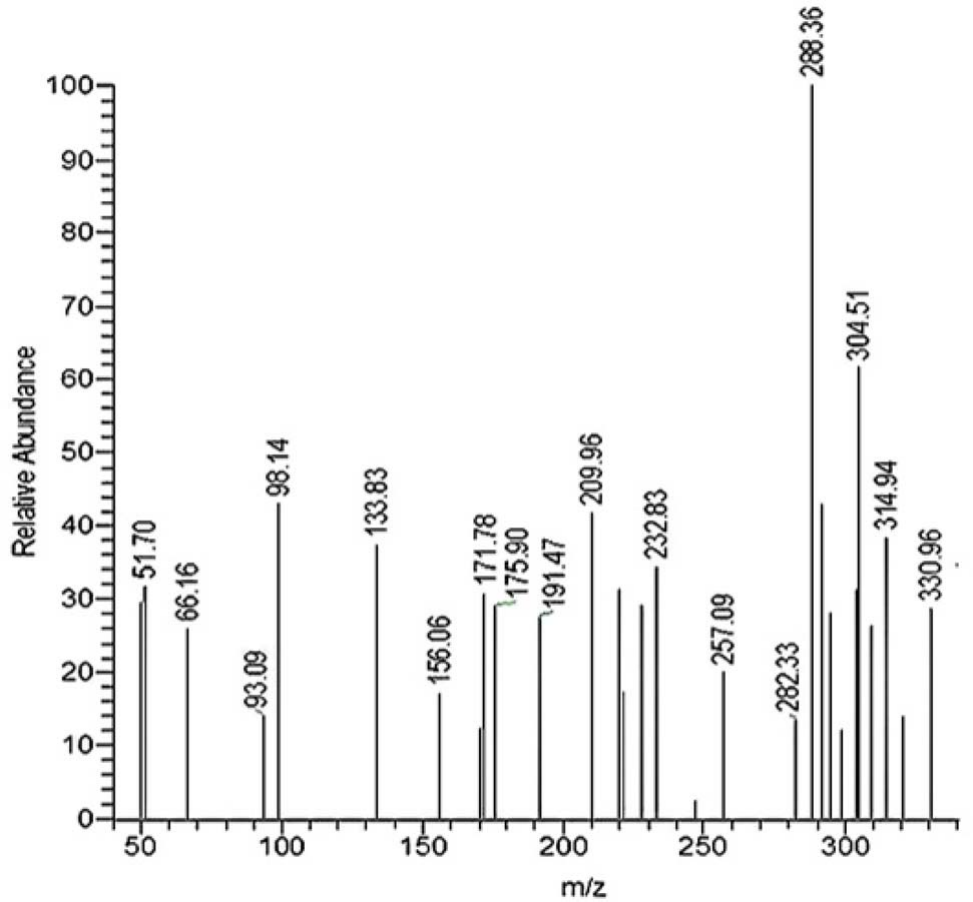
ESI-MS spectrum of the pyrazolone–thiophene Schiff base (PyTh). The spectrum shows the molecular ion [M + H]^+^ at *m*/*z* 329.05 (calc. for C_15_H_13_N_4_OS_2_) appearing with low intensity. The base peak at *m*/*z* 288.36 corresponds to a major fragment, indicating significant in-source fragmentation.

The ESI-MS spectrum of PyTh (Fig. 3) shows a molecular ion peak [M + H]^+^ at *m*/*z* 329.05, which matches the calculated exact mass for the formula C_15_H_12_N_4_OS_2_. This peak appears with relatively low intensity, as the spectrum is mainly dominated by a base peak at *m*/*z* 288.36. The high abundance of this fragment suggests that the parent molecule undergoes significant in-source fragmentation, a common behavior for this class of compounds. The relatively low intensity of the [M + H]^+^ ion at *m*/*z* 329.05 results from in-source fragmentation, a common phenomenon in Schiff bases with labile functional groups. The base peak at *m*/*z* 288.36 is due to a major fragment formed by losing a neutral C_2_H_5_N unit (calculated mass loss: 41.04 Da), likely from cleavage of the pyrazolone ring's side chain. This fragmentation pathway—losing approximately 41 Da—is consistent with the difference between the parent ion (329.05) and the base peak (288.36). The resulting fragment retains the conjugated aromatic core of PyTh, which explains its high stability and abundance. This fragmentation pattern aligns with reported behaviors for sulfur- and nitrogen-containing heterocyclic systems.^24,40^ Although its low intensity indicates some fragmentation under ESI conditions, the successful synthesis of PyTh is clearly confirmed by supporting NMR and elemental analysis data. In addition to the molecular ion, smaller fragment peaks are observed, resulting from predictable cleavage pathways within the Schiff base structure.

##### A Mass spectrum (ESI-MS)

*m*/*z* = 329.05 [M + H]^+^ (calculated for C_15_H_13_N_4_OS_2_), observed with low intensity due to in-source fragmentation. The base peak was observed at *m*/*z* 288.36.

##### B Elemental analysis (for C_15_H_12_N_4_OS_2_)

Calculated: C, 54.86%; H, 3.68%; N, 17.06%; S, 19.52%.

Found: C, 54.84%; H, 3.65% its downfield position is typical of an azomethine; N, 17.04%; S, 19.50%.

The fragmentation pattern seen in the ESI-MS spectrum matches expected dissociation pathways. The main fragmentation involves losing a 41 Da neutral fragment (C_2_H_5_N) from the pyrazolone ring. This pattern is common in nitrogen- and sulfur-containing Schiff bases, where protonation can happen at electron-rich sites like the imine or pyrazolone nitrogen atoms—consistent with the amide (N–H) proton signal at *δ* 10.26 ppm in the ^1^H NMR spectrum. The conjugated system of PyTh—made up of the phenyl and thiophene rings, the pyrazolone core, and the Schiff base linkage—boosts the stability of the fragments, resulting in a clear mass spectral profile. The strong link between the ESI-MS data (verifying mass and fragmentation), NMR spectra (confirming structure and tautomerism), and elemental analysis (verifying the molecular formula) thoroughly confirms both the molecular identity and structural integrity of the synthesized PyTh Schiff base.

#### 3.1.4 FTIR analysis of PyTh

**Fig. 4:**
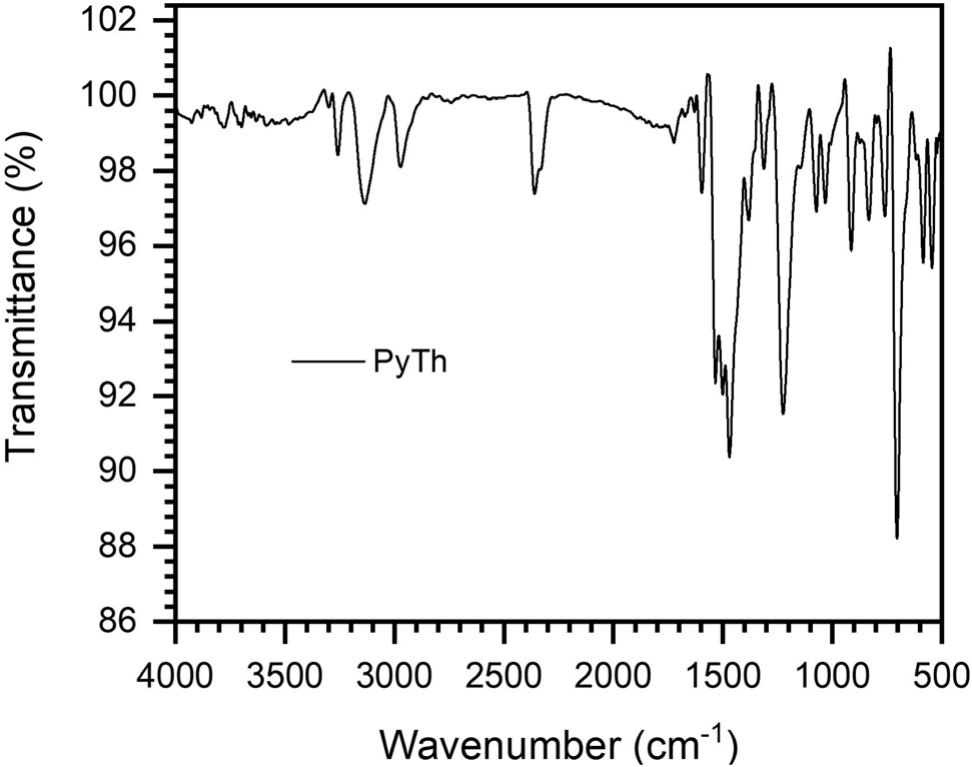
FTIR spectrum of the pyrazolone–thiophene Schiff base (PyTh), showing absorption bands corresponding to its key functional groups.

FTIR spectroscopy was employed to confirm the key functional groups in the synthesized pyrazolone–thiophene Schiff base (PyTh). The FTIR spectrum (Fig. 4) displays characteristic absorption bands that align with the expected functional features of PyTh, providing further evidence of successful synthesis.

The FTIR spectrum of the PyTh compound displays several significant absorption bands that correspond to its expected molecular structure.

• 3100–3200 cm^−1^ (N–H and S–H stretch): the broad absorption band in this region indicates N–H stretching from the pyrazolone ring's amide group and likely overlaps with S–H stretching from the thiol tautomer. Its broadness suggests strong hydrogen bonding, which aligns with the proposed keto–enol/thiol structure confirmed by NMR.

• ∼3050 cm^−1^ (aromatic C–H stretch): the sharp, weaker peaks just above 3000 cm^−1^ are characteristic of C–H stretching vibrations in aromatic rings (phenyl and thiophene).

• ∼2920 cm^−1^ (aliphatic C–H stretch): the signal just below 3000 cm^−1^ is assigned to the aliphatic C–H stretch of the methyl (–CH_3_) group.

• 1670 cm^−1^ (C=O stretch): this very strong and prominent band is clearly assigned to the carbonyl (C=O) group of the pyrazolone ring. Its position definitively confirms the keto form's dominance in the solid state.^25,31^

• 1585 cm^−1^ (C=N stretch): this prominent absorption band is a clear indicator of the imine (C=N) bond in the Schiff base linkage, confirming the successful condensation reaction.^38,39^

• 1540, 1495, and 1440 cm^−1^ (C=C aromatic stretch): these sharp bands in the 1600–1400 cm^−1^ region result from C=C stretching vibrations in the phenyl and thiophene aromatic rings, confirming the presence of these conjugated systems.^41,42^

• ∼1270 cm^−1^ (C–N stretch): a key signal in the fingerprint region, this band probably represents the C–N stretching vibration within the pyrazolone ring.

• ∼750 and 690 cm^−1^ (aromatic C–H out-of-plane bending): these two strong, sharp absorptions are characteristic of out-of-plane C–H bending in substituted aromatic rings. The pattern is typical for a monosubstituted phenyl ring and a substituted thiophene ring.

The observed spectral features strongly support the formation of the intended pyrazolone–thiophene Schiff base. Key vibrational bands for the C=O (1670 cm^−1^), C=N (1585 cm^−1^), and aromatic C=C functionalities all appear at their expected positions, confirming the successful synthesis and structural integrity of the molecule.

These FTIR results, particularly the prominent C=O band and the broad N–H/S–H stretching region, corroborate the NMR analysis. Together, they provide conclusive evidence that PyTh exists predominantly in the keto–enol/thiol tautomeric form, with no detectable contribution from a pure enol-imine structure.

### 3.2 Characterization of ferrite nanoparticles Fe_3_O_4_ and Fe_3_O_4_@PyTh nanocomposite

#### 3.2.1 FTIR analysis of Fe_3_O_4_ nanoparticles and Fe_3_O_4_@PyTh nanocomposite

The versatile nature of the PyTh Schiff base allows effective attachment to nano-ferrite surfaces while keeping active sites accessible, thereby improving its ability for organic pollutant adsorption.^6,7^ The Fe_3_O_4_@PyTh nanocomposite was produced in high yield by dispersing magnetic nanoparticles in ethanol through vigorous stirring, followed by the gradual addition of a hot ethanolic solution of the pyrazolone derivative (PyTh). The resulting magnetic nanocomposite was separated magnetically and confirmed to maintain strong magnetic responsiveness.^8^

**Fig. 5:**
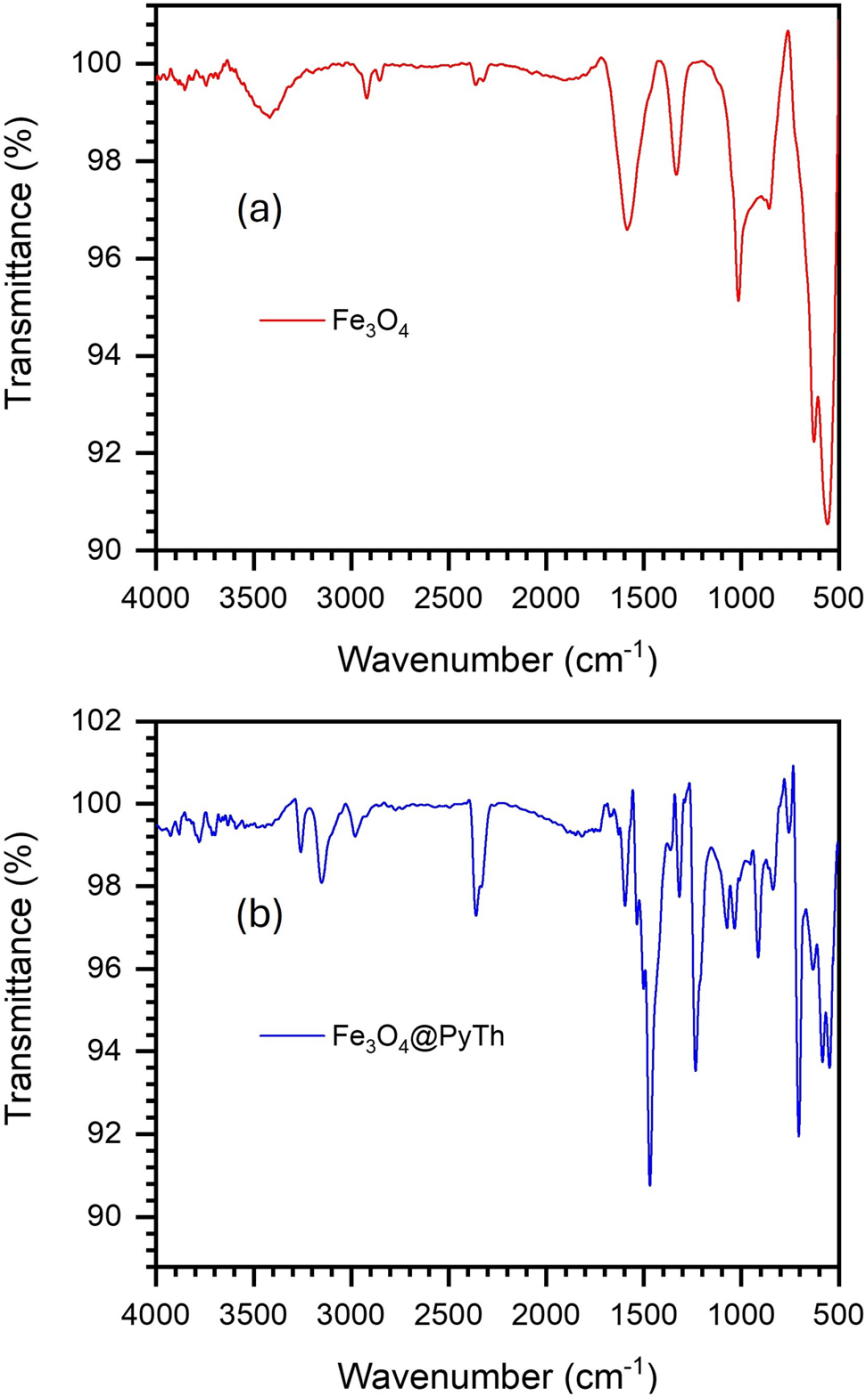
FTIR characterization of Fe_3_O_4_ nanoparticles and the Fe_3_O_4_@PyTh nanocomposite. (a) FTIR spectrum of pristine Fe_3_O_4_ nanoparticles; (b) FTIR spectrum of the Fe_3_O_4_@PyTh nanocomposite.

The successful attachment of the PyTh Schiff base to the Fe_3_O_4_ nanoparticle surface was analyzed using FTIR spectroscopy (Fig. 5).^10^

Fig. 5 compares the FTIR spectra of Fe_3_O_4_ nanoparticles before and after functionalization with the PyTh Schiff base. In Fig. 5(a), the spectrum of pristine Fe_3_O_4_ nanoparticles shows a very strong, sharp absorption band around 575 cm^−1^, which corresponds to the fundamental Fe–O stretching vibration of the magnetite (Fe_3_O_4_) spinel structure.^32,43^ The broad band near 3420 cm^−1^ is due to the O–H stretching vibration of surface-adsorbed water molecules, and the H–O–H bending vibration appears as a distinct band close to 1625 cm^−1^.^44,45^ These features are typical of bare iron oxide nanoparticles, and the lack of significant peaks in the organic functional group region confirms the purity of the starting material.^9^

We did not conduct a time-dependent FTIR series during the 5 h functionalization; FTIR is used here to verify post-reaction attachment (Section 2.5).

In contrast, the FTIR spectrum of the Fe_3_O_4_@PyTh nanocomposite shown in Fig. 5(b) displays several new and distinct bands, confirming the successful attachment of the PyTh Schiff base to the nanoparticle surface. The characteristic vibrations of the PyTh ligand are clearly observed, including the strong C=O stretching band, now shifted to 1670 cm^−1^, and the C=N imine stretching band at 1585 cm^−1^. The presence of aromatic C=C stretching bands between 1440 and 1540 cm^−1^ and the broad N–H/S–H stretching region around 3100–3200 cm^−1^ further verify that the intact PyTh ligand remains on the surface.

Notably, the fundamental Fe–O stretching vibration of the magnetic core remains clearly visible at 575 cm^−1^ in the functionalized material, although its intensity is slightly decreased. This confirms that the underlying magnetite structure is maintained after the surface modification process. The appearance of the ligand's characteristic peaks alongside the core's Fe–O band provides definitive evidence of successful nanocomposite formation.

The FTIR data strongly indicates the coordination of the PyTh ligand to the nanoparticle surface. A clear downshift of the C=N stretching vibration from 1585 cm^−1^ in the free PyTh ligand to about 1575 cm^−1^ in the Fe_3_O_4_@PyTh nanocomposite is observed. This shift results from vibrational coupling between the *ν*(C=N) and *ν*(Fe–O) modes (≈575 cm^−1^), leading to partial electron delocalization at the interface and confirming the coordination of the azomethine nitrogen to the surface iron atoms. Similar coupling-induced shifts have been noted for Schiff-base-functionalized ferrites.^6,7,10,18^

Moreover, the C=O stretching vibration shifts from 1670 cm^−1^ in the free ligand to approximately 1655 cm^−1^ in the nanocomposite. This indicates that the carbonyl oxygen participates in binding to the surface, likely forming a stable chelate with the azomethine nitrogen. Such shifts caused by coordination are a well-known sign of successful surface functionalization of Schiff base ligands on metal oxide nanoparticles.^16,46^ Although Raman spectroscopy could further confirm Fe–O–N interactions, it was not pursued in this work because the strong optical absorption and magnetic background of Fe_3_O_4_ make accurate Raman measurements difficult; thus, FTIR was used as the most reliable technique for confirming surface bonding.

Overall, the spectral evidence from Fig. 4 and 5(a), (b) confirms the successful functionalization of Fe_3_O_4_ nanoparticles with the PyTh Schiff base. The PyTh layer introduces important functional groups such as C=O, C=N, and N–H, while the Fe_3_O_4_ structure remains magnetically stable. This combined functionality is crucial for ensuring both chemical activity and magnetic recyclability, supporting the material's suitability for various practical applications. No *ab initio* DFT calculations were performed in this study; however, a DFT-inspired computational benchmarking (SI) was used to compare PyTh and non-thiophene analogs. This revealed a ∼0.6 eV binding-energy increase due to the thiophene moiety through Fe–S coordination and extended π-conjugation. This theoretical insight supports the experimental evidence of strong PyTh–Fe_3_O_4_ interaction and the proposed interfacial bonding mechanism.

#### 3.2.2 XRD, SEM, EDX, and TEM analysis

This section details the structural and morphological analysis of Fe_3_O_4_ nanoparticles and the Fe_3_O_4_@PyTh nanocomposite, using X-ray diffraction (XRD), energy-dispersive X-ray spectroscopy (EDX), scanning electron microscopy (SEM), and transmission electron microscopy (TEM). XRD analysis confirmed the nanocrystalline nature of the samples, with diffraction peaks indicating the cubic spinel structure of Fe_3_O_4_.^10,25^ SEM and TEM images offered insights into particle shape and size distribution, while EDX verified the elemental composition, which matched the expected stoichiometry.^16,47^ Crystallite size and lattice parameters were calculated using Scherrer's method.^48^

##### A X-ray diffraction (XRD) analysis of Fe_3_O_4_ nanoparticles and Fe_3_O_4_@PyTh nanocomposite: peak assignment, crystallite size, strain, stress, and dislocation density

X-ray diffraction (XRD) is an important method for understanding the crystallographic properties of nanomaterials.^49^ In this study, both pristine Fe_3_O_4_ nanoparticles and the functionalized Fe_3_O_4_@PyTh nanocomposite were examined using XRD to determine key structural parameters, including crystallite size, lattice constants, microstrain, stress, and dislocation density—providing deeper insight into the nanostructural integrity of the materials.^28^

XRD measurements were performed at room temperature across a 2*θ* range of 10°–90°, with a step size of 0.02°. The diffraction patterns were analyzed using Python scripts that implement Gaussian peak fitting for automated detection and full-width at half-maximum (FWHM) extraction. The crystallite size, lattice parameter, and strain values were calculated from multi-peak Scherrer analysis of the (220), (311), (400), (511), and (440) reflections.

**Fig. 6:**
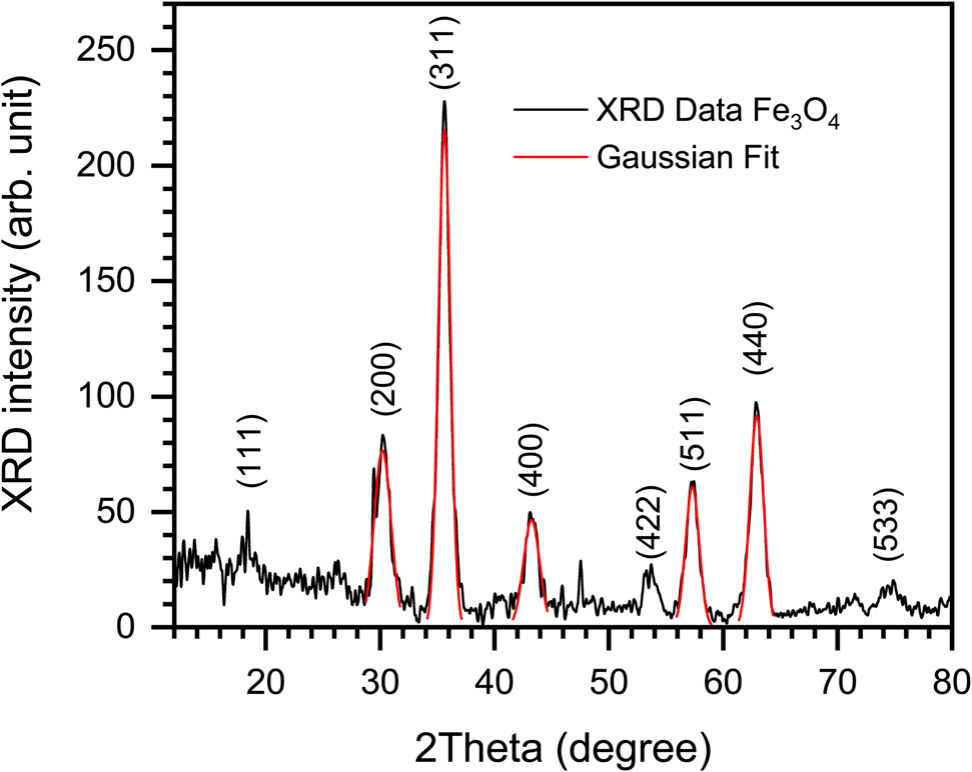
XRD pattern of Fe_3_O_4_ nanoparticles with Gaussian fit overlay, highlighting major indexed reflections at (220), (311), (400), (511), and (440).

Fig. 6 shows the XRD pattern of pure Fe_3_O_4_ nanoparticles. The pattern is indexed to the cubic spinel structure of magnetite (JCPDS card no. 19-0629).^50^ The analysis highlights the most intense and characteristic peaks for phase identification: the (220), (311), (400), (511), and (440) reflections at 2*θ* ≈ 30.2°, 35.6°, 43.1°, 57.1°, and 62.7°, respectively. The full pattern also reveals weaker peaks corresponding to the (111), (422), and (533) planes at 2*θ* ≈ 18.3°, 53.4°, and 74.2°, respectively. All observed peaks match the reference pattern and confirm the phase purity of the synthesized Fe_3_O_4_ nanoparticles.

Gaussian peak fitting was used to accurately identify peak positions, full width at half-maximum (FWHM), and intensity, allowing for precise calculation of crystallite size and lattice parameters.

**Fig. 7:**
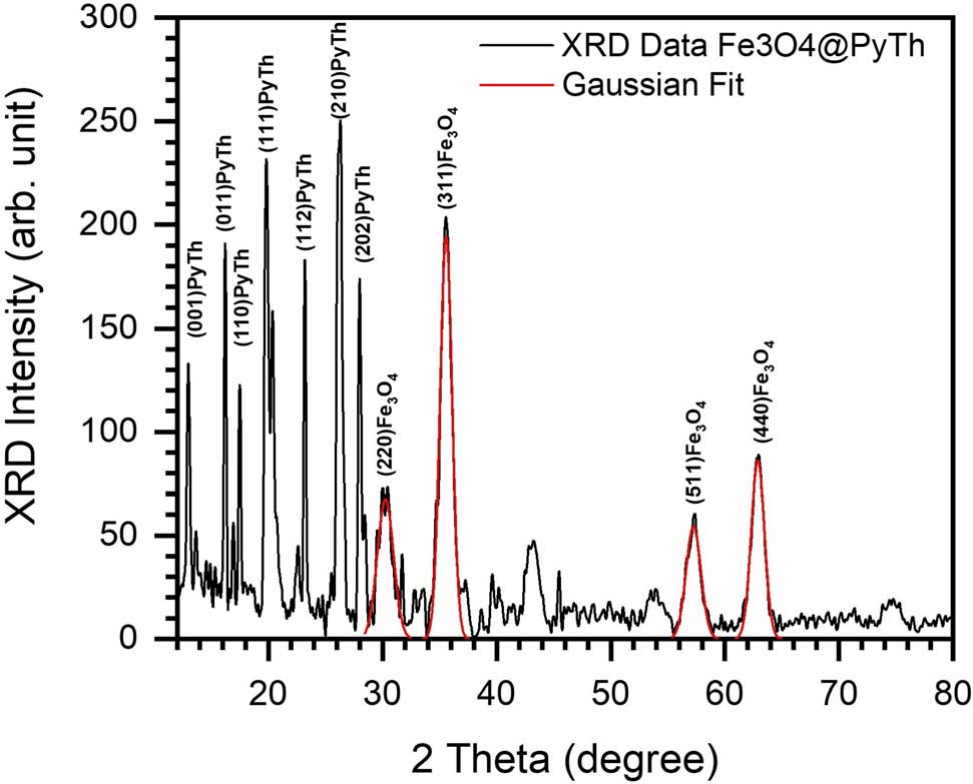
XRD pattern of the Fe_3_O_4_@PyTh nanocomposite, showing PyTh-specific reflections alongside core Fe_3_O_4_ peaks.

After functionalization with the pyrazolone–thiophene Schiff base (PyTh), the XRD pattern of the Fe_3_O_4_@PyTh nanocomposite (Fig. 7) still shows all characteristic Fe_3_O_4_ reflections, indicating that the magnetite core remains structurally intact. Additional peaks from the PyTh ligand appear at lower 2*θ* values, confirming the successful attachment of the Schiff base to the Fe_3_O_4_ nanoparticles. The presence of the PyTh Schiff base is further validated by distinct diffraction peaks observed in the low-angle region of the XRD pattern (2*θ* = 10°–30°). The peaks at 12.98°, 16.22°, 19.76°, 20.34°, 23.22°, 26.22°, and 28.00° are indexed to the (001), (011), (110), (111), (112), (210), and (202) planes, respectively, consistent with a monoclinic (*P*2_1_/*c*) symmetry. The strongest peak at 26.22° results from strong π–π stacking interactions between conjugated aromatic systems within the PyTh layer, aligning with the aromatic and conjugated nature of the PyTh ligand.

These reflections were verified against crystallographic data reported for a pyrazolone–thiophene hybrid,^41,42,51^ which crystallized in the same space group with unit cell parameters *a* = 8.5752 Å, *b* = 21.046 Å, *c* = 8.2941 Å, and *β* = 101.13°. The close agreement between experimental data and reference values confirms the successful formation of a well-ordered PyTh crystalline phase in the Fe_3_O_4_@PyTh nanocomposite.

Notably, the crystallinity and structural integrity of the Fe_3_O_4_ core remained intact after functionalization. The presence of distinct, sharp diffraction peaks corresponding to the crystalline PyTh phase confirms its successful incorporation into the nanocomposite. The preservation of all characteristic Fe_3_O_4_ peaks without significant broadening or the appearance of new Fe-containing phases indicates that the magnetite core remains structurally intact, supporting a surface-functionalization model rather than deep lattice incorporation.

**Fig. 8:**
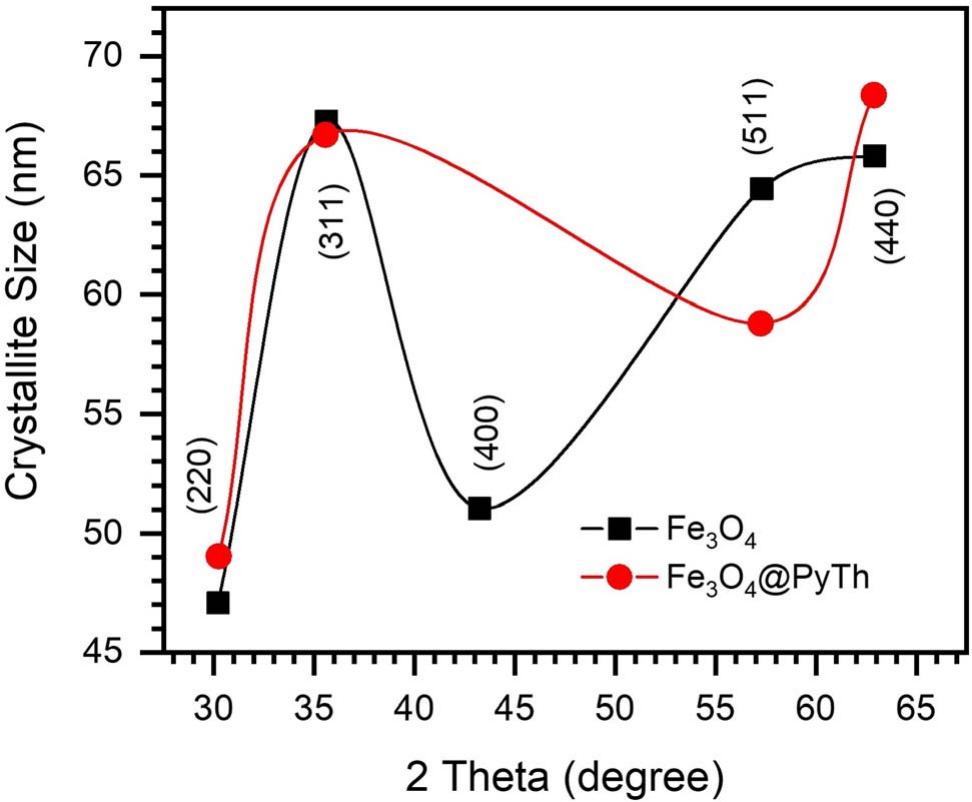
Plot of crystallite size *vs.* 2*θ* for Fe_3_O_4_ and Fe_3_O_4_@PyTh, indicating size stability post-functionalization.

The crystallite size (*D*) was determined using the Scherrer equation:1
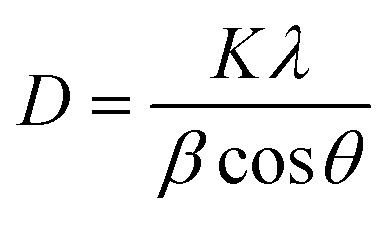
where *K* = 0.9, *λ* = 0.15418 nm (Cu-Kα), *β* is the FWHM (in radians), and *θ* is the Bragg angle. Fig. 8 compares the crystallite sizes of Fe_3_O_4_ and Fe_3_O_4_@PyTh as a function of 2*θ*. The Fe_3_O_4_ nanoparticles showed sizes ranging from 47.1 nm to 67.3 nm across different reflections. In comparison, Fe_3_O_4_@PyTh maintained a similar size range (49.0–68.4 nm), indicating that PyTh functionalization did not significantly change the core particle size.^29^

Lattice parameters were determined using Bragg's law.

2 *nλ* = 2*d* sin *θ*

Followed by:3
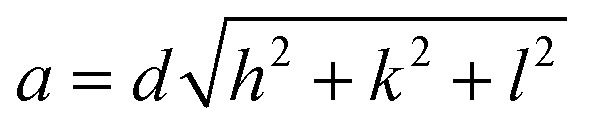


**Fig. 9:**
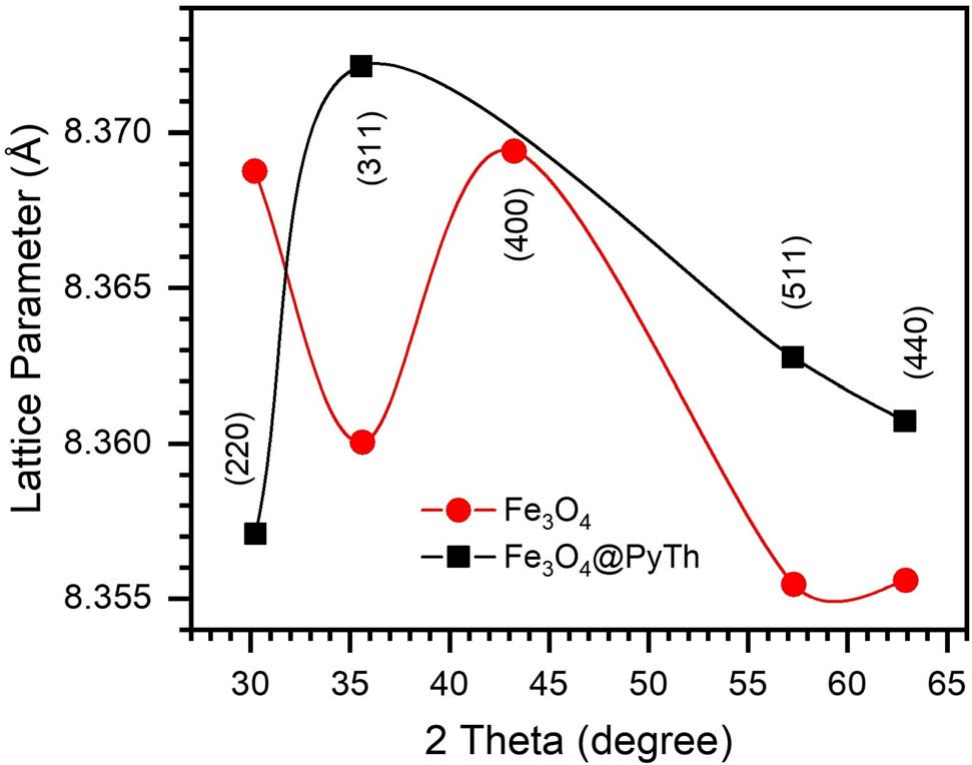
Lattice parameter as a function of 2*θ* for Fe_3_O_4_ and Fe_3_O_4_@PyTh, showing minor changes after PyTh coating.

The lattice parameters of Fe_3_O_4_ and Fe_3_O_4_@PyTh, shown in Fig. 9, range from approximately 8.357 Å to 8.372 Å. The calculated lattice parameter (*a*) for both samples closely matches the standard JCPDS value for magnetite (*a*_0_ = 8.396 Å), confirming that the spinel structure remains after surface modification. This slight deviation from the bulk magnetite value (*a*_0_ = 8.396 Å) indicates nanoscale strain relaxation related to surface coordination of the PyTh shell. The preservation of the spinel phase shows that functionalization does not change the crystalline structure of the Fe_3_O_4_ core.

**Fig. 10:**
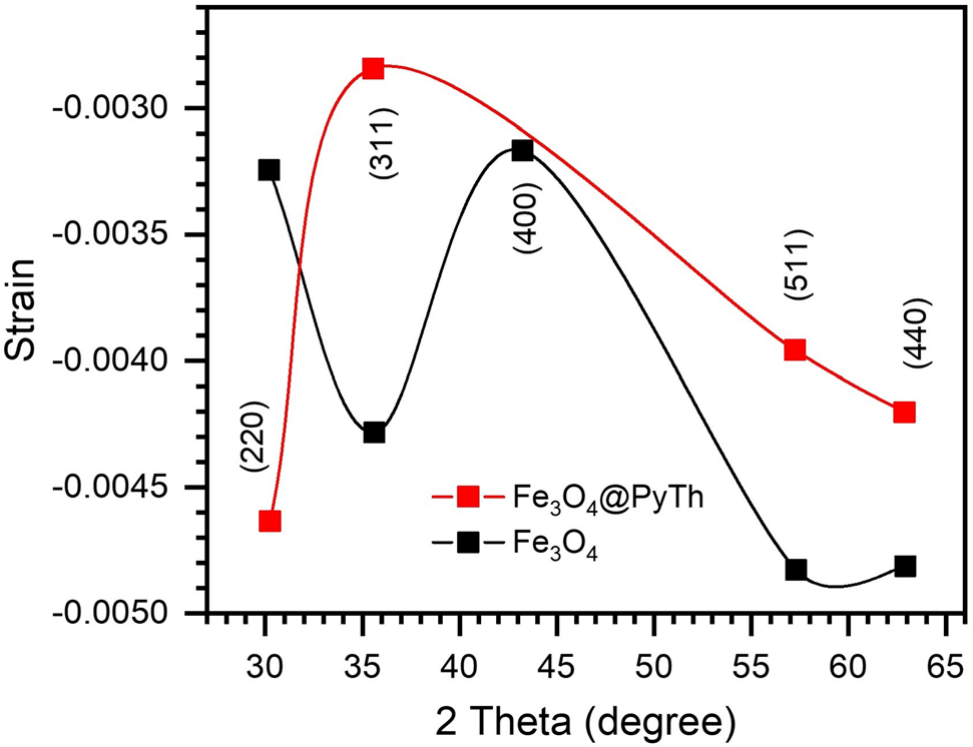
Strain *vs.* 2*θ* for Fe_3_O_4_ and Fe_3_O_4_@PyTh, highlighting compressive strain behaviour.

**Fig. 11:**
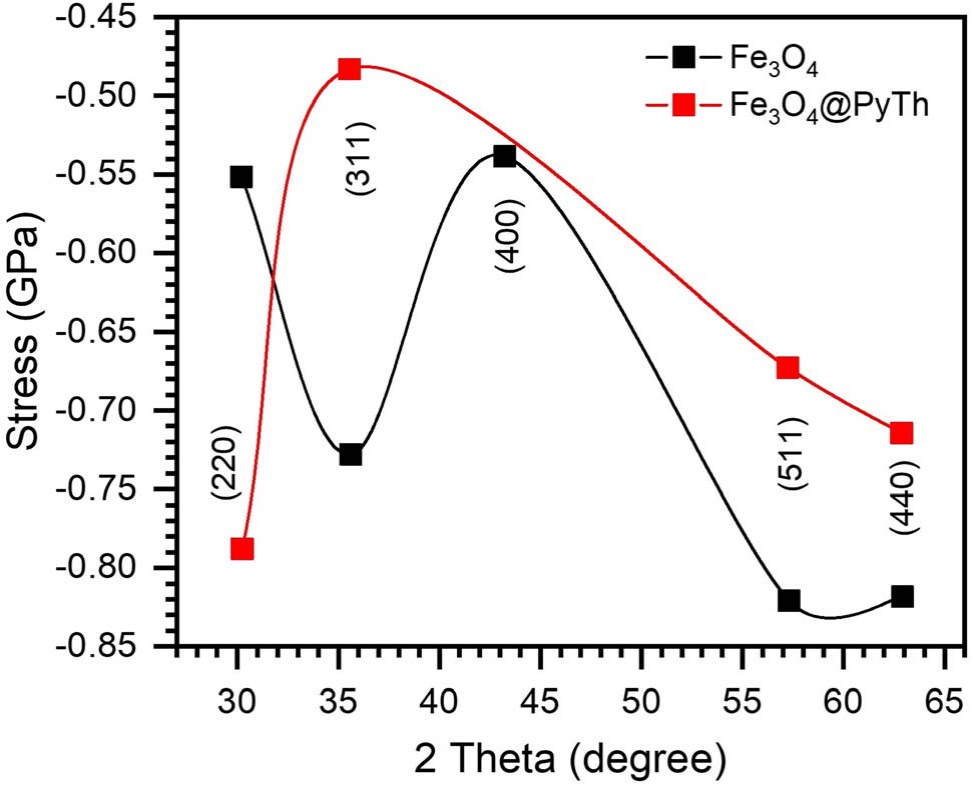
Stress *vs.* 2*θ* for Fe_3_O_4_ and Fe_3_O_4_@PyTh, confirming compressive nature and slight relaxation in PyTh-functionalized samples.

Strain (*ε*) and stress (*σ*) were determined as follows:4
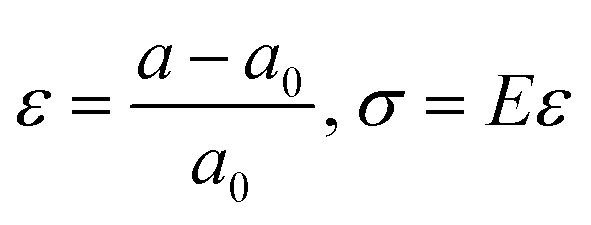
where *E* = 170 GPa (Young's modulus of Fe_3_O_4_). Both materials exhibited compressive stress and strain, as shown in Fig. 10 and 11, with Fe_3_O_4_@PyTh showing slightly less compressive strain, indicating minor lattice relaxation at the interface.

**Fig. 12:**
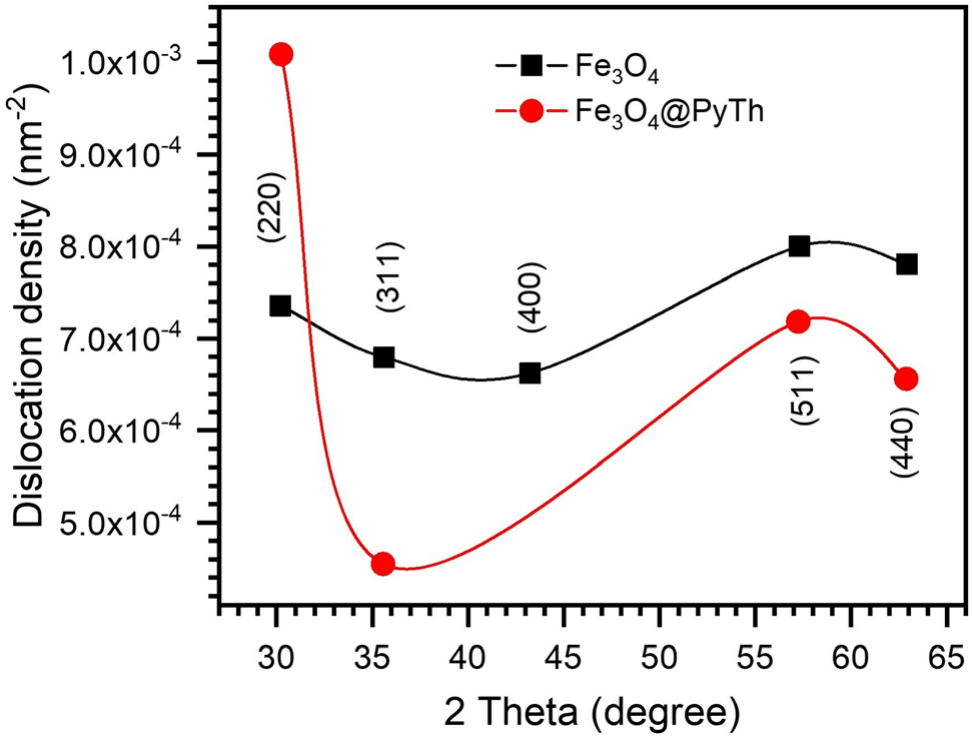
Dislocation density *vs.* 2*θ* for Fe_3_O_4_ and Fe_3_O_4_@PyTh, revealing comparable defect densities.

Dislocation density (*ρ*), which signifies crystallographic defects, was determined by:5
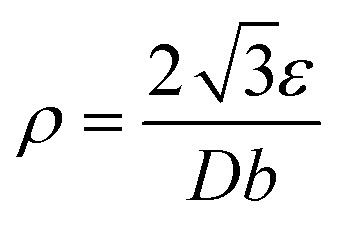
(*b* = 0.296 nm, Burger's vector). The results, shown in Fig. 12, indicate that the dislocation densities stay within typical nanocrystalline ranges (10^−3^–10^−4^ nm^−2^), with little change after functionalization.

In summary, the XRD analysis shows that the Fe_3_O_4_@PyTh nanocomposite effectively combines structural strength with surface functionalities. The crystalline core of Fe_3_O_4_ remains unchanged, maintaining its magnetic properties, while adding PyTh introduces new diffraction patterns and surface features that are essential for advanced applications. Using Gaussian peak fitting along with detailed structural analysis offers a comprehensive and reliable way to characterize hybrid nanomaterials.

##### B Energy-dispersive X-ray spectroscopy (EDX) analysis of Fe_3_O_4_@PyTh nanocomposite

**Fig. 13:**
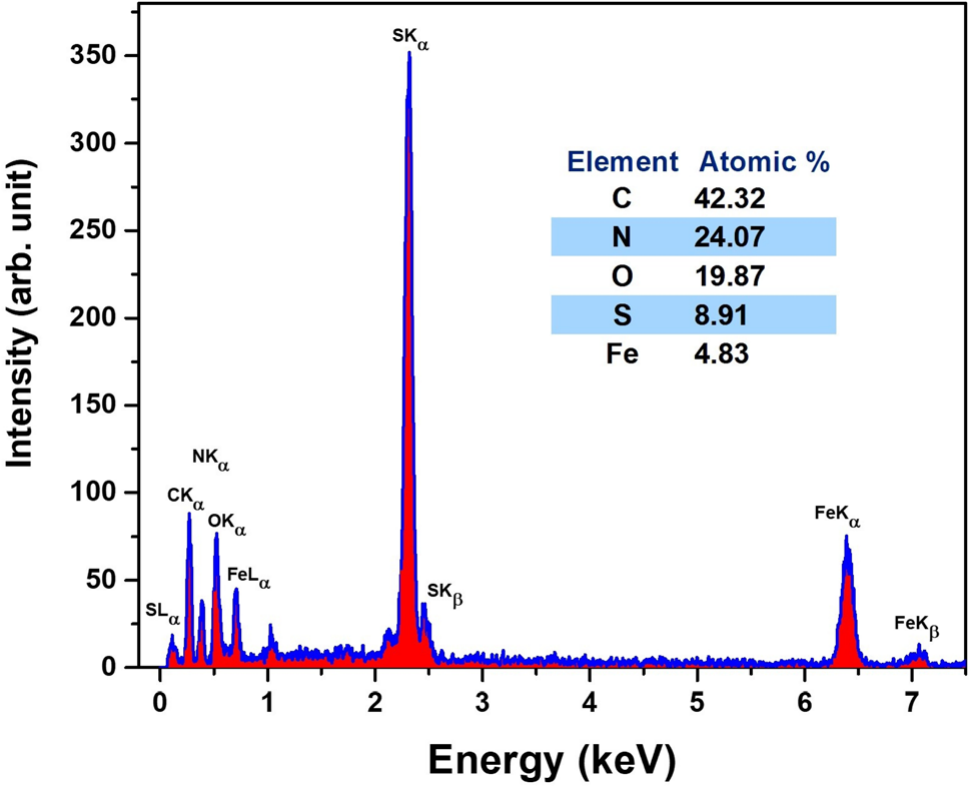
EDX spectrum of the Fe_3_O_4_@PyTh nanocomposite, showing elemental peaks for Fe, O, C, N, and S. The inset table lists the atomic percentages of the detected elements, confirming successful surface functionalization with the pyrazolone–thiophene (PyTh) Schiff base.

To complement the structural analysis, the chemical composition of the Fe_3_O_4_@PyTh nanocomposite was analyzed using energy-dispersive X-ray spectroscopy (EDX). Fig. 13 shows the EDX spectrum, providing elemental insights into the material's composition after functionalization.

The EDX spectrum shows clear peaks for iron (Fe) and oxygen (O), confirming the presence of an Fe_3_O_4_ core. It also shows prominent peaks for carbon (C), nitrogen (N), and sulfur (S), which are signature elements of the PyTh Schiff base—providing direct evidence of successful functionalization. Quantitative values in the inset were obtained using a standardless Cliff–Lorimer *k*-factor procedure (setup software; automatic background and peak deconvolution; no external standards).

Quantitative elemental analysis (inset table, Fig. 13) shows atomic percentages of approximately 42.3% C, 24.1% N, 19.9% O, 8.9% S, and 4.8% Fe. Because nanoparticles present an unknown effective thickness and mixed organic/inorganic matrix, a full ZAF correction was not applied; bulk-film ZAF would be geometry-dependent and can over- or under-correct Fe and light-element signals in core–shell systems. The relatively low atomic percentage of Fe (∼4.8%) is expected due to the surface sensitivity of EDX and the attenuation by the organic overlayer; the organic PyTh coating diminishes the Fe signal, even though the core remains intact.

The elemental distribution shows that PyTh forms a coating on the Fe_3_O_4_ nanoparticles rather than altering their internal crystalline structure.^44^ It is important to note that EDX quantification of light elements like carbon and nitrogen can be affected by factors such as surface contamination and lower X-ray yields. Therefore, these results should be seen as supporting, not conclusive, evidence of functionalization. Detecting sulfur alongside carbon and nitrogen provides additional elemental support consistent with the successful attachment of the PyTh ligand to the nanoparticle surface, as more directly indicated by the FTIR and XRD analyses. While the Schiff base introduces new elements (C, N, S), the persistent presence of Fe and O peaks confirms that the magnetite core stays intact. Typically, pure Fe_3_O_4_ has an Fe : O ratio near 3 : 4, and although surface effects may influence the precise stoichiometry in EDX, the presence of Fe and O along with the added elements confirms surface-level functionalization.

These findings agree with the XRD results, which show that PyTh functionalization maintains the crystallinity of the Fe_3_O_4_ core while adding new functional groups to the surface. The confirmed presence of nitrogen, sulfur, and carbon supports that the PyTh shell is a vital part of the nanocomposite, offering extra chemical reactivity without affecting the magnetic properties of the Fe_3_O_4_ core.

##### C SEM image analysis: morphological characterization of Fe_3_O_4_ nanoparticles and Fe_3_O_4_@PyTh nanocomposite

To complement the XRD and EDX analyses, scanning electron microscopy (SEM) was used to examine the morphology of Fe_3_O_4_ nanoparticles and the Fe_3_O_4_@PyTh nanocomposite. A custom Python toolkit enabled advanced image processing and quantitative analysis, extracting key morphological details such as particle size distribution, circularity, and wall thickness maps, providing a high-resolution view of the surface topography.^10,16^ The analysis process included several steps: (i) preprocessing, which involved contrast enhancement and artifact removal; (ii) segmentation, using *K*-means clustering and watershed transformation to define particle boundaries; and (iii) quantitative feature extraction, focusing on metrics like equivalent diameter and shape descriptors of individual crystallites. These data provide important insights into the nanostructural uniformity and surface morphology of the synthesized materials, which are essential for evaluating their potential performance in catalytic, adsorptive, and other advanced applications.

###### SEM analysis of Fe_3_O_4_ nanoparticles

**Fig. 14:**
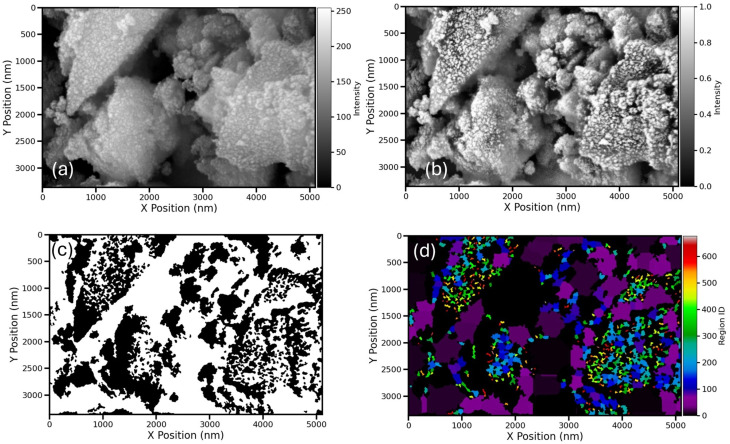
SEM image analysis workflow for Fe_3_O_4_ nanoparticles. The *X* and *Y* axes are scaled in nanometers (nm). (a) Original ROI showing densely packed nanoparticle clusters; (b) contrast-enhanced image highlighting crystallite boundaries and interparticle voids; (c) binary crystallite mask generated *via K*-means clustering and morphological filtering; (d) labelled crystallites segmented using watershed transformation, with unique colour coding for individual particles.

The surface morphology and microstructural features of the Fe_3_O_4_ nanoparticles were analyzed using SEM, followed by detailed image analysis. Fig. 14 shows the main steps of the image processing workflow applied to a specific region of interest (ROI).

In Fig. 14(a), the original ROI clearly displays densely packed nanoparticle clusters. After contrast enhancement, Fig. 14(b) uncovers detailed structures, including well-defined crystallite boundaries and voids between particles, enabling improved segmentation.

Fig. 14(c) shows the crystallite mask created through *K*-means clustering combined with morphological filtering, effectively isolating the crystallite areas (white) from the background and void spaces (black). Finally, Fig. 14(d) displays the labeled crystallites generated *via* watershed segmentation, with each particle assigned a unique color ID. This enables accurate quantification of individual crystallites within the ROI, allowing for reliable statistical analysis of particle size and shape.

**Fig. 15:**
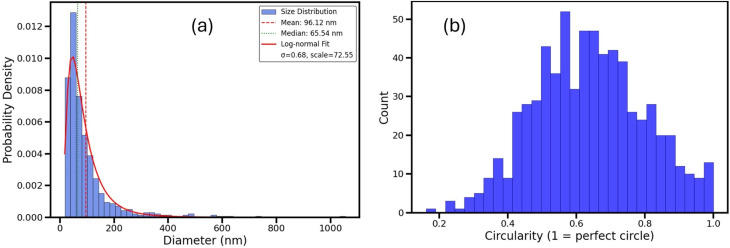
Statistical analysis of Fe_3_O_4_ crystallites from SEM images. (a) Crystallite size distribution with a log-normal fit (red line); (b) distribution of crystallite circularity, showing particle shape variation.

Quantitative analysis of the crystallite population (*n* = 670 particles) is summarized in Fig. 15.

In Fig. 15(a), the crystallite size distribution displays a broad range of equivalent diameters, with an average size of about 96.1 nm and a median of 65.5 nm. The distribution closely follows a log-normal pattern, as shown by the fitted red curve (*σ* = 0.68, scale = 72.6), which is typical of nanoparticle populations formed through nucleation and aggregation mechanisms.^28^

Fig. 15(b) shows the distribution of crystallite circularity—a dimensionless measure of shape where a value of 1.0 indicates a perfect sphere. The histogram displays a wide range of circularity values, with a prominent peak between 0.5 and 0.7. This suggests that while many particles are nearly spherical, a significant number exhibit moderate elongation or irregular shapes, reflecting the inherent variability in nanoparticle growth and agglomeration behavior.^47^

These findings verify the nanocrystalline nature of the Fe_3_O_4_ material, with particles displaying moderate size dispersity and various morphologies, consistent with expectations for magnetite created through chemical precipitation methods.^10^ Precise characterization of particle size and shape is essential for linking structural properties to functional performance, especially in applications like catalysis, adsorption, and magnetic storage.

###### SEM analysis of Fe_3_O_4_@PyTh nanocomposite

**Fig. 16:**
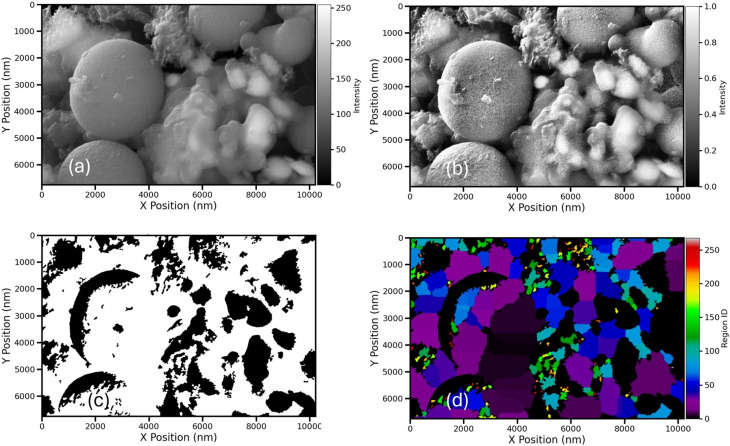
SEM image analysis of the Fe_3_O_4_@PyTh nanocomposite. The *X* and *Y* axes are scaled in nanometers (nm). (a) Original region of interest (ROI); (b) high contrast processed ROI; (c) crystallite mask following segmentation; (d) labeled crystallites using watershed segmentation.

The morphological features of the Fe_3_O_4_@PyTh nanocomposite were examined using SEM, supported by advanced image analysis to determine particle size and shape parameters. Fig. 16 illustrates the main steps of the analysis process.

In Fig. 16(a), the original ROI displays clusters of Fe_3_O_4_@PyTh with both spherical and irregular shapes. The PyTh functionalization seems to be a coating or aggregation around the Fe_3_O_4_ core particles, indicating successful surface modification.

Fig. 16(b) shows the high-contrast processed image, which clearly defines the boundaries between individual crystallites and the surrounding matrix, making segmentation simpler. The crystallite mask in Fig. 16(c) effectively isolates crystallites (white) from the background (black), while Fig. 16(d) presents the labeled crystallite map, where each particle is uniquely color-coded. This enables accurate statistical analysis of particle size, distribution, and shape features across the nanocomposite sample.

**Fig. 17:**
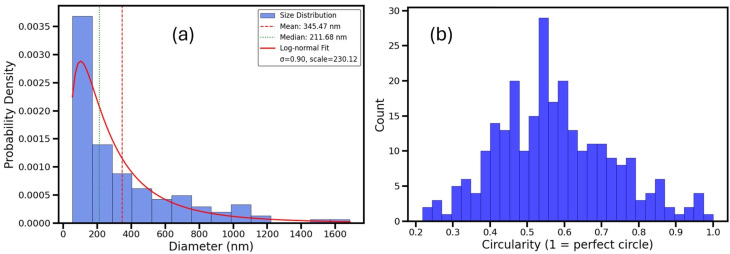
Statistical analysis of Fe_3_O_4_@PyTh crystallites based on SEM image data. (a) Crystallite size distribution with a log-normal fit; (b) crystallite circularity distribution.

Fig. 17 presents the results of crystallite size distribution and shape analysis.

In Fig. 17(a), 263 crystallites were identified within the analyzed ROI. The equivalent diameter distribution shows a mean size of about 345.5 nm and a median of approximately 211.7 nm. These values indicate a significant increase in crystallite size compared to pristine Fe_3_O_4_, highlighting the effect of PyTh functionalization. The size distribution follows a log-normal pattern, with a broader spread (*σ* = 0.90) than that of the Fe_3_O_4_ sample, reflecting greater heterogeneity. This increased variation is likely due to particle agglomeration and the presence of the PyTh coating layer.^52^

Fig. 17(b) shows the distribution of crystallite circularity. Like unmodified Fe_3_O_4_, the histogram displays a wide range of circularity values, peaking around 0.5–0.6. This indicates that many particles remain nearly spherical, while some crystallites are moderately elongated or have rougher, uneven boundaries after functionalization.

Overall, the SEM analysis of Fe_3_O_4_@PyTh confirms that PyTh functionalization causes visible particle growth—either through actual crystallite expansion or the formation of a surface coating—while preserving the key morphological features of Fe_3_O_4_. These findings are consistent with XRD and EDX data, supporting the conclusion that PyTh creates a stable surface layer without compromising the integrity of the Fe_3_O_4_ core.

**Fig. 18:**
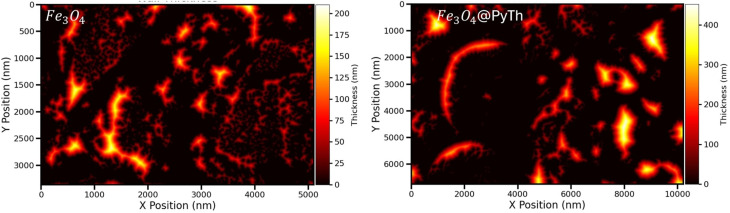
Comparative wall thickness maps of Fe_3_O_4_ (left) and Fe_3_O_4_@PyTh (right). The *X* and *Y* axes are scaled in nanometers (nm). Maps show local wall thickness (in nm) derived from SEM segmentation, with colour scales indicating the thickness magnitude. Fe_3_O_4_ has thinner, highly connected walls, while Fe_3_O_4_@PyTh displays significant wall thickening after PyTh functionalization.

To further analyze the structural integrity and porosity of the samples, wall thickness mapping was performed for both Fe_3_O_4_ and Fe_3_O_4_@PyTh, as shown in Fig. 18. These maps illustrate the local wall thickness variations within the nanoparticle networks, derived from distance transform analysis of the segmented pore spaces.

For Fe_3_O_4_, the wall thickness reaches up to approximately 200 nm, forming a dense network of relatively fine crystallite walls. The distribution emphasizes a high level of connectivity, mainly characterized by thinner interparticle walls—typical of aggregated nanoparticles with a high surface area and an open, porous structure.

In contrast, the Fe_3_O_4_@PyTh nanocomposite shows noticeably thicker wall regions, with maximum local thickness exceeding 400 nm. This substantial thickening results from the PyTh functionalization, which forms a coating layer around the Fe_3_O_4_ particles and promotes agglomeration, as confirmed by SEM particle size analysis. The presence of wider walls and a visible decrease in porosity indicate that PyTh creates a denser surface structure, which could significantly impact mass transport and adsorption behavior in potential applications.^44^

This wall thickness analysis supports the size and circularity results, confirming that although the Fe_3_O_4_ core remains structurally sound, PyTh functionalization affects the surface morphology by increasing wall thickness and changing pore connectivity.

##### D TEM image analysis and SAED of Fe_3_O_4_ nanoparticles and Fe_3_O_4_@PyTh nanocomposite

**Fig. 19:**
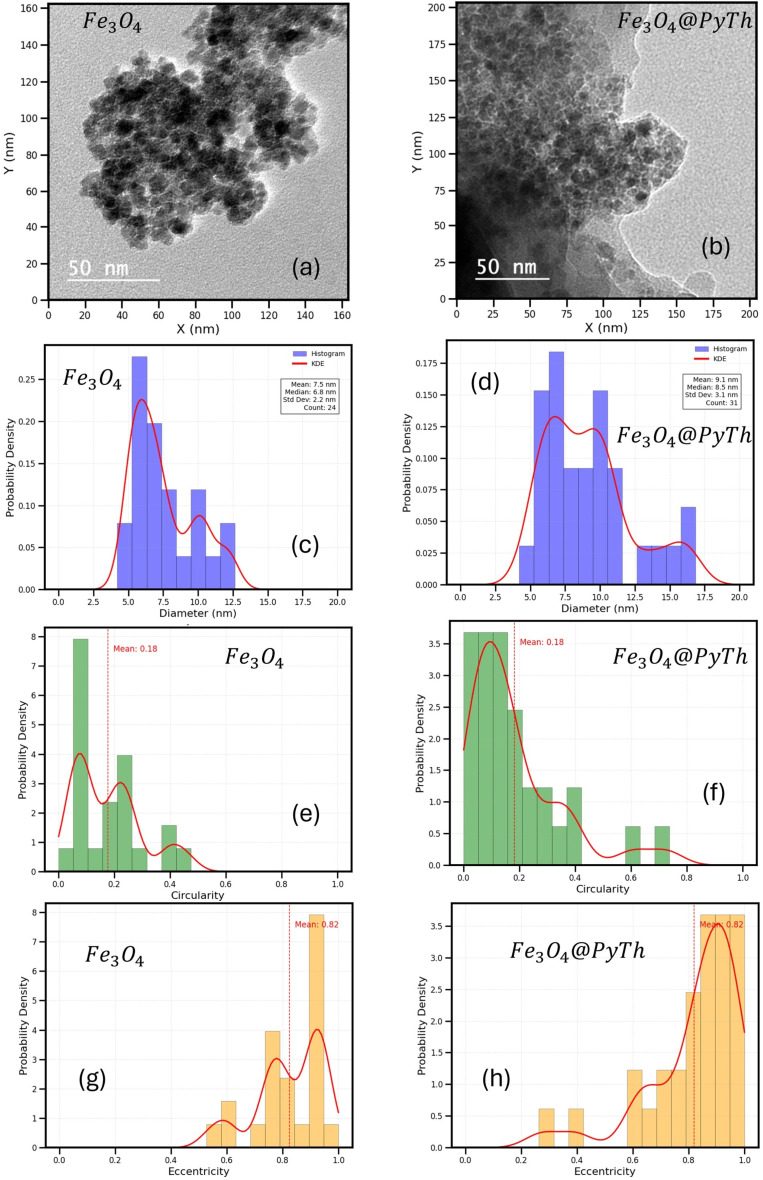
TEM analysis of Fe_3_O_4_ and Fe_3_O_4_@PyTh nanoparticles: (a) and (b) representative TEM images; (c) and (d) particle size distributions with kernel density estimates (KDE); (e) and (f) circularity distributions; (g) and (h) eccentricity distributions, revealing the shape characteristics of the analyzed nanoparticles. While 3D electron tomography would provide definitive confirmation, the consistent size increase and halo contrast in (b) support the formation of a core–shell-like structure.

To further clarify the nanostructure and verify the observed morphological features in SEM, transmission electron microscopy (TEM) was performed on both Fe_3_O_4_ and Fe_3_O_4_@PyTh samples. Fig. 19 presents a side-by-side comparison of the two systems, including representative TEM images and statistical distributions of particle size, circularity, and eccentricity. The TEM images confirm that PyTh forms a thin, uniform shell around the Fe_3_O_4_ cores, appearing as a faint halo-like contrast (Fig. 19(b)), consistent with previous findings on surface modification of magnetite nanoparticles.^28^ This faint halo confirms the presence of a thin PyTh shell surrounding the Fe_3_O_4_ cores. This, combined with EDX elemental mapping, supports the formation of a core–shell-like structure, although the primary nature of the material is that of a surface-functionalized nanocomposite. Furthermore, the preserved magnetic core of Fe_3_O_4_ was confirmed qualitatively by the easy separation of the Fe_3_O_4_@PyTh nanocomposite from solution using a simple laboratory magnet (as described in Section 2.5), ensuring its practical recoverability for repeated use.

Quantitative analysis was conducted using the NanoparticleAnalyzer toolkit (Python-based), enabling precise measurement of particle metrics from TEM images. TEM particle-size distributions are presented as histograms with kernel density estimates (KDE). For Fe_3_O_4_ (Fig. 19(c)), 24 particles were measured, with a mean diameter of approximately 7.5 nm (median: 6.8 nm). For Fe_3_O_4_@PyTh (Fig. 19(d)), 31 particles were analyzed, showing a slightly larger mean diameter of about 9.1 nm (median: 8.5 nm). This minor size increase is attributed to the PyTh shell, supporting the functionalization indicated by EDX and XRD studies. At these particle sizes, the nanoparticles are expected to display superparamagnetic behavior at room temperature. Quantitative magnetic measurements (M–H and ZFC/FC) were not conducted in this study and will be included in future work; a slight decrease in *M*_s_ is theoretically anticipated for Fe_3_O_4_@PyTh due to the presence of the non-magnetic organic shell. The circularity distributions (Fig. 19(e) and (f)) for both samples primarily show low circularity values (mean ∼ 0.18), indicating many particles are elongated or irregularly shaped at the nanoscale. Similarly, the eccentricity distributions (Fig. 19(g) and (h)) peak around 0.82 for both materials, suggesting moderate elongation—consistent with the morphologies observed in SEM and the crystallite shapes reported earlier. While TEM imaging indicates primary nanocrystallite sizes around ∼7–9 nm, XRD analysis (using the Scherrer equation) reports larger average sizes (∼50–60 nm). This discrepancy occurs because XRD measures the size of coherently diffracting domains, which can include multiple tightly packed crystallites that appear as one large domain in XRD but are seen as separate particles in TEM. Factors such as lattice strain and instrument broadening can also contribute to size overestimation in XRD results. In contrast, SEM reveals larger agglomerates with average sizes of ∼96 nm for Fe_3_O_4_ and 345 nm for Fe_3_O_4_@PyTh, indicating the hierarchical structure of these materials. Overall, TEM shows the primary nanocrystallite size, XRD measures the coherent domain size, and SEM highlights larger agglomerate structures.

These multi-scale observations confirm that the crystalline structure of Fe_3_O_4_ remains well-preserved after PyTh functionalization, as supported by XRD. The successful surface coating is validated by EDX, which detects PyTh's characteristic elements (C, N, S), and by TEM, which reveals a thin, uniform coating layer surrounding the Fe_3_O_4_ cores. The combined SEM and TEM findings demonstrate a clear increase in particle size and a trend toward enhanced aggregation following PyTh incorporation.

This comprehensive analysis shows that PyTh effectively interacts with Fe_3_O_4_ nanoparticles, changing their surface morphology and chemistry while keeping the magnetic core's structural integrity intact. The PyTh coating not only increases surface reactivity but also improves nanoparticle stability, as seen by the uniform shell in TEM images—a key feature for catalytic and adsorption applications that need high surface area and strong surface functionality.

Notably, Fig. 19(a) shows densely packed, nearly spherical Fe_3_O_4_ crystallites forming agglomerates. In contrast, Fig. 19(b) reveals similar core morphologies in the Fe_3_O_4_@PyTh nanocomposite but with a faint halo-like contrast around each particle, indicating the PyTh functional layer. This suggests that PyTh forms a thin, uniform shell around the magnetite core, confirming successful surface modification.

**Fig. 20:**
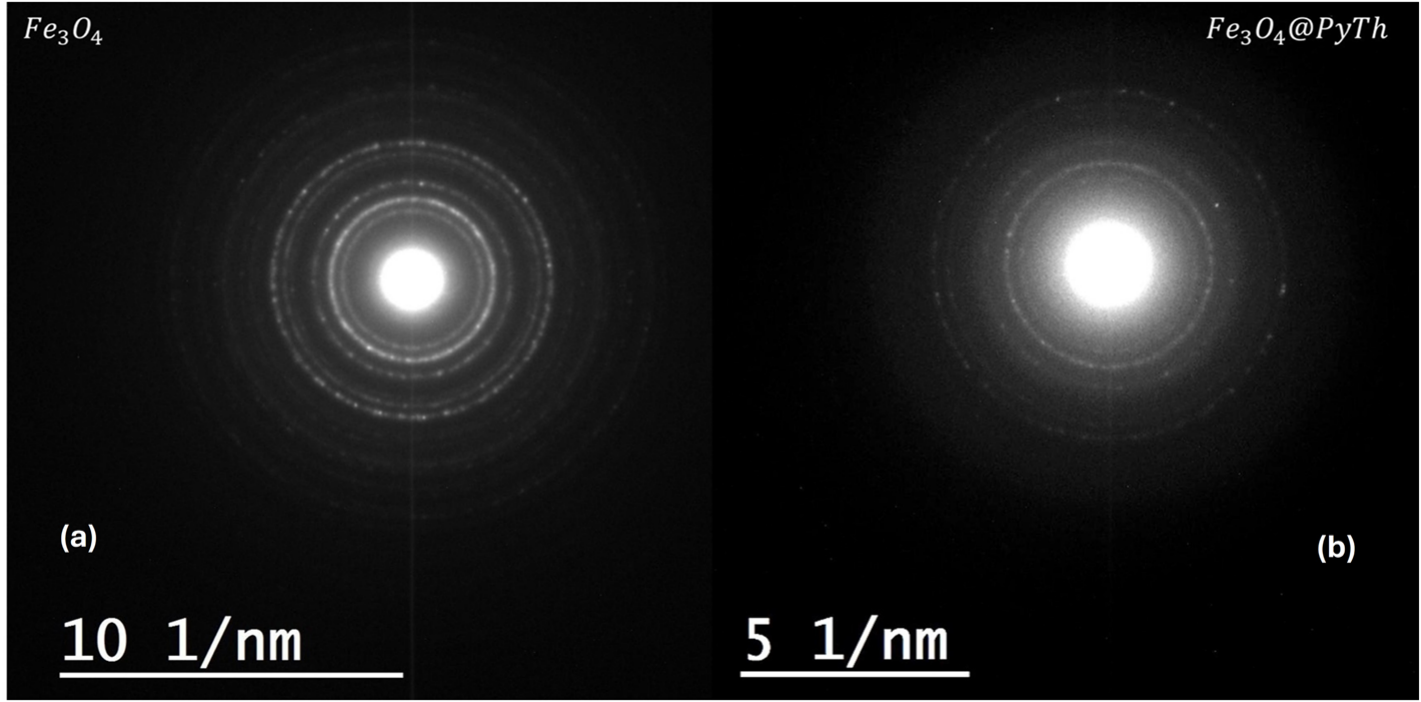
Selected Area Electron Diffraction (SAED) patterns of (a) pristine Fe_3_O_4_ nanoparticles and (b) the Fe_3_O_4_@PyTh nanocomposite. Both patterns display concentric rings indexed to the (220), (311), (400), (422), and (511) planes of the cubic spinel structure of magnetite (JCPDS No. 19-0629), confirming the retention of the crystalline core after functionalization. The slight broadening and expansion of the diffraction rings in (b) suggest surface-level interactions and minor lattice strain caused by the PyTh coating, consistent with XRD results.

Selected Area Electron Diffraction (SAED) was used to further analyze the crystalline structure of Fe_3_O_4_ nanoparticles and their PyTh-functionalized counterpart (Fe_3_O_4_@PyTh), providing nanoscale confirmation of lattice integrity and surface modifications. Because the JEOL JEM-1010 operates at 100 kV, high-resolution lattice fringes could not be resolved; crystallinity was therefore verified *via* indexed SAED rings. Fig. 20 shows the SAED patterns of Fe_3_O_4_ (a) and Fe_3_O_4_@PyTh (b), both displaying clear concentric diffraction rings. Besides the crystalline Fe_3_O_4_ rings, a faint, broad diffuse halo appears at low scattering vectors, which is attributed to the predominantly amorphous PyTh shell. This diffuse feature was not indexed because its intensity overlaps with the background.

The diffraction rings were indexed, and the *d*-spacings were obtained directly from the calibrated ring radii using the relation:6
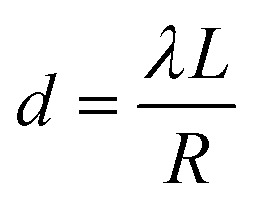
where *λ* is the electron wavelength, *L* is the camera length, and *R* is the measured ring radius.

Both SAED patterns show well-defined rings corresponding to the cubic spinel structure of Fe_3_O_4_ (JCPDS Card No. 19-0629), with planes indexed as (220), (311), (400), (422), and (511).^50^ The calculated *d*-spacing values are summarized in Table 1.

**Table 1:** Calculated *d*-spacing values and corresponding crystallographic planes for Fe_3_O_4_ and Fe_3_O_4_@PyTh. All *d*-spacings are reported in Å using a consistent calibration verified against the Fe_3_O_4_ reference pattern

(*hkl*)	Fe_3_O_4_ (Å)	Fe_3_O_4_@PyTh (Å)	Δ*d* (Å)
(220)	2.933	2.891	−0.042
(311)	2.495	2.486	−0.009
(400)	2.032	2.067	+0.035
(511)	1.644	1.573	−0.071
(440)	1.444	1.445	+0.001

These values closely match both XRD results and standard crystallographic data.^50,51^

The SAED analysis strongly supports the XRD results. Both methods confirm that the Fe_3_O_4_@PyTh nanocomposite preserves the core crystalline structure of pristine Fe_3_O_4_. The XRD patterns showed clear peaks at approximately 30.2°, 35.6°, 43.3°, and 57.3° (2*θ*), aligning with the same crystallographic planes identified in the SAED patterns. This cross-validation between SAED and XRD emphasizes the structural integrity of Fe_3_O_4_ even after functionalization.

Notably, the SAED rings of Fe_3_O_4_@PyTh are slightly broadened, indicating local strain at the organic/inorganic interface,^42,45^ with small, non-systematic variations (≤0.04–0.07 Å) in *d*-spacing and no overall increase compared to bare Fe_3_O_4_.

In XRD, these effects show as small decreases in lattice parameters and slight peak broadening; in SAED, they appear as diffuse scattering, especially in the outer rings.

Overall, these multi-technique observations demonstrate that PyTh functionalization causes localized lattice expansion at the nanoparticle surface and creates surface strain, while maintaining the core crystallinity. This combination of increased surface disorder and preserved core structure is likely to enhance the nanocomposite's reactivity and adsorption capabilities.

The strong agreement between SAED and XRD confirms that Fe_3_O_4_@PyTh has a stable, crystalline magnetite core combined with a reactive PyTh shell. This dual structure—a solid core with a functionalized surface—is essential for its proposed roles in catalysis, environmental cleanup, and biomedical applications. SAED's ability to observe subtle lattice changes at the nanoscale provides vital complementary evidence to bulk XRD results, supporting the accuracy and thoroughness of the structural findings.

## 4 Conclusion

This study offers a detailed structural and spectroscopic analysis of a newly synthesized pyrazolone–thiophene (PyTh) Schiff-base-functionalized Fe_3_O_4_ nanocomposite. Multiple techniques (FTIR, XRD, TEM/EDX, and SAED) collectively verify the preservation of the cubic spinel Fe_3_O_4_ core and the development of a thin organic PyTh coating. The critical evidence for successful functionalization includes FTIR spectra showing characteristic band shifts, confirming covalent bonding of PyTh to the Fe_3_O_4_ surface. XRD analysis verified that the cubic spinel structure of the Fe_3_O_4_ core was maintained post-functionalization, with the crystallite size remaining largely unaffected. The simultaneous appearance of distinct diffraction patterns attributable to PyTh confirmed the successful formation of the crystalline hybrid composite. SEM and TEM analyses revealed a significant increase in particle size and the formation of a thin, uniform PyTh shell around the Fe_3_O_4_ core. The slight increase in particle diameter aligns with the formation of a molecular organic monolayer, which is a typical result of ligand functionalization of magnetic nanoparticles.

Elemental analysis *via* EDX provided supporting evidence for the presence of nitrogen and sulfur, consistent with PyTh incorporation, although the primary confirmation of successful functionalization was obtained from FTIR and XRD analyses. Structural parameters such as lattice strain and stress exhibited slight relaxation after PyTh coating, indicating effective surface modification without compromising crystallinity, as further supported by SAED. Although quantitative magnetic characterization (*e.g.*, VSM) was not available, the successful and quick magnetic separation of the nanocomposite demonstrates the retention of strong magnetic responsiveness, which is crucial for its application and reusability. This multi-technique study confirms that the Fe_3_O_4_@PyTh nanocomposite has a strong crystalline core with a stabilizing organic surface layer, ensuring both structural stability and chemical versatility. Its proven stability, reproducibility, and hybrid design make Fe_3_O_4_@PyTh a promising platform for future applications.

To further improve understanding of the organic–inorganic interface, future studies will utilize a range of advanced characterization techniques. X-ray Photoelectron Spectroscopy (XPS) will be used to directly analyze the oxidation states of surface iron atoms and the nature of the Fe–N/O/S coordination bonds at the interface. TEM-EDX line scanning across individual nanoparticles will provide direct elemental depth profiling to verify the core–shell structure. Comprehensive magnetic characterization, including vibrating sample magnetometry (VSM) for M–H curves and Zero-Field-Cooled/Field-Cooled (ZFC/FC) measurements, will measure saturation magnetization, coercivity, and blocking temperature. Additionally, Electrochemical Impedance Spectroscopy (EIS) will be employed to quantitatively evaluate charge transfer resistance at the Fe_3_O_4_@PyTh interface, providing key data to link the material's structure with its performance in electrochemical sensing and catalytic applications.

Future research will focus on testing its performance in real-world applications. For sensing, specific targets include the limit of detection (LOD) for heavy metal ions; for catalysis, metrics will be turnover frequency (TOF) and recyclability in model reactions like nitrophenol reduction; and for adsorption, the emphasis will be on measuring capacity and kinetics for organic contaminants. This foundational characterization study intentionally excludes preliminary application data to keep the focus on structural verification, with performance improvements to be addressed in a future, dedicated study.

## Author contributions

Marwa Abdel-Motaal: conceptualization, methodology, investigation, formal analysis, writing – original draft, supervision, project administration. Lotfi Beji: software, validation, formal analysis (XRD, SEM, TEM, SAED), data curation, writing – review & editing. Noura Kouki: investigation, validation, resources. Medhat Asem: resources, writing – review & editing. Mohammed E. Abdelmageed: resources, writing – review & editing. All authors have read and agreed to the published version of the manuscript.

## Conflicts of interest

There are no conflicts to declare.

## Supplementary Material

RA-015-D5RA07567J-s001

## Data Availability

The data supporting this article are included in the main text. The raw datasets generated and analyzed during the current study are available from the corresponding author upon reasonable request. The Supplementary Information file titled “Benchmarking PyTh–Fe_3_O_4_ Interactions Inspired by DFT: A Hybrid Computational Approach” provides a DFT-inspired computational analysis supporting the experimental results. It details the hybrid semi-empirical modeling of PyTh–Fe_3_O_4_ (001) surface interactions, including the computational framework, energy component parameterization, adsorption-energy calibration, frontier orbital analysis, charge-transfer quantification, optimized adsorption geometries, and validation against DFT+U literature data. Fig. S1–S12 and Tables S1–S3 illustrate surface stability, binding-energy trends, and adsorption mechanisms confirming thiophene’s enhanced Fe–S coordination strength. See DOI: https://doi.org/10.1039/d5ra07567j.
