# Supplementary material for: Novel pyrazolone–thiophene Schiff base functionalized Fe3O4 nanocomposite: core–shell structure and multi-technique characterization
Source: RSC Adv. 2025 Nov 7;15(50):43120–40. doi: 10.1039/d5ra07567j (PMC12593418; doi:10.1039/d5ra07567j)
Supplement: RA-015-D5RA07567J-s001 [file RA-015-D5RA07567J-s001.pdf]

Supplementary Information (SI) for RSC Advances.
This journal is © The Royal Society of Chemistry 2025

Supplementary File

Benchmarking PyTh–Fe_3O_4 Interactions Inspired by DFT: A Hybrid Computational Approach

Purpose

This supplementary note provides a DFT-inspired computational analysis to support the experimental findings in the main paper. A hybrid, semi-empirical model—parameterized with established DFT data—was developed to assess the interaction between the pyrazolone–thiophene Schiff base (PyTh) ligand and the Fe_3O_4 (001) surface. The model clarifies why PyTh's affinity is higher compared to its non-thiophene counterparts and links fundamental ab initio understanding with computational efficiency. The methodology and calibration follow the framework of established studies,[1,2] ensuring physically realistic adsorption-energy scaling.

1. Computational Framework and Theoretical Model

A hybrid approach that combines DFT-parameterized bond-energy expressions, electrostatic and dispersion interactions, and charge-transfer stabilization was implemented to model the complex Fe–ligand interface.

1.1 Surface Model

The Fe_3O_4 (001) facet was chosen because it is the most stable and commonly exposed orientation in magnetite nanoparticles.[3,4] This surface has alternating layers of Fe^{2+} and Fe^{3+} cations with high densities of coordinatively unsaturated sites capable of multidentate binding. A 4 × 4 supercell slab (lattice parameter = 3.0 Å) was used, based on the DFT-optimized structure reported by Roldan et al.[1]

1.2 Total Binding Energy Expression

The total binding energy (E_{bind}) is calculated by summing the individual contributions:

$$E_{\text{bind}} = E_{\text{coord}} + E_{\text{electrostatic}} + E_{\text{vdW}} + E_{\text{CT}} \quad \text{(Eq.S1)}$$

where the terms respectively refer to coordination bonding, Coulombic interaction, dispersion, and charge-transfer stabilization. All reported magnitudes are model scores (arbitrary units, a.u.); a calibrated scale (Section 11) converts them into realistic adsorption energies (eV).

1.3 Energy Component Parameterization

(A) Coordination Energy (E_{coord})

Modeled using a combination of a DFT-inspired Gaussian term and a Morse potential for covalent bonding.

$$E_{\text{bond}} = E_{\text{DFT}} e^{-\alpha(r - r_{\text{opt}})^2} - D_e[1 - e^{-\alpha(r - r_e)}]^2 \quad \text{(Eq.S2)}$$

with $\alpha = 2.0$ Å^{-2}.

Interaction	E_{DFT} (eV)	r_{opt} (Å)	D_e (eV)	α (Å^{-1})	r_e (Å)	Ref.
Fe–S	–1.8	2.3	1.8	1.8	2.3	1
Fe–N	–1.2	2.1	1.2	2.0	2.1	5
Fe–O	–1.5	2.0	1.5	2.2	2.0	5

These parameters replicate the energy hierarchy Fe–S > Fe–O > Fe–N observed in DFT+U calculations.[1, 5]

(B) Electrostatic Energy ($E_{\mathrm{electrostatic}}$)

$$E = k\frac{q_1 q_2}{r}, k = 14.4 \qquad \text{(Eq.S3)}$$

Partial charges (e) from population analysis[6]: S = –0.45, N = –0.35, O = –0.55, Fe = +0.65.

(C) Van der Waals Energy (E_{vdW})

$$E = 4\varepsilon\left[\left(\frac{\sigma}{r}\right)^{12} - \left(\frac{\sigma}{r}\right)^{6}\right] \qquad \text{(Eq.S4)}$$

Pair	ε (eV)	σ (Å)	Ref.
Fe–S	0.25	2.3	7
Fe–N	0.15	2.1	7
Fe–O	0.20	2.0	7
Fe–C	0.08	2.5	7
Fe–H	0.02	2.8	7

(D) Charge-Transfer Energy (E_{CT})

$$E_{CT} = -\frac{1}{2}\Delta q(\varepsilon_{HOMO} - \Phi) \quad \text{(Eq.S5)}$$

Δq = charge transferred (e); Φ = 5.2 eV (work function of Fe_3O_4 (001)).[8] Transfer values: Fe–S = 0.30 e, Fe–N = 0.20 e, Fe–O = 0.25 e.[2]

2. Surface Selection Justification

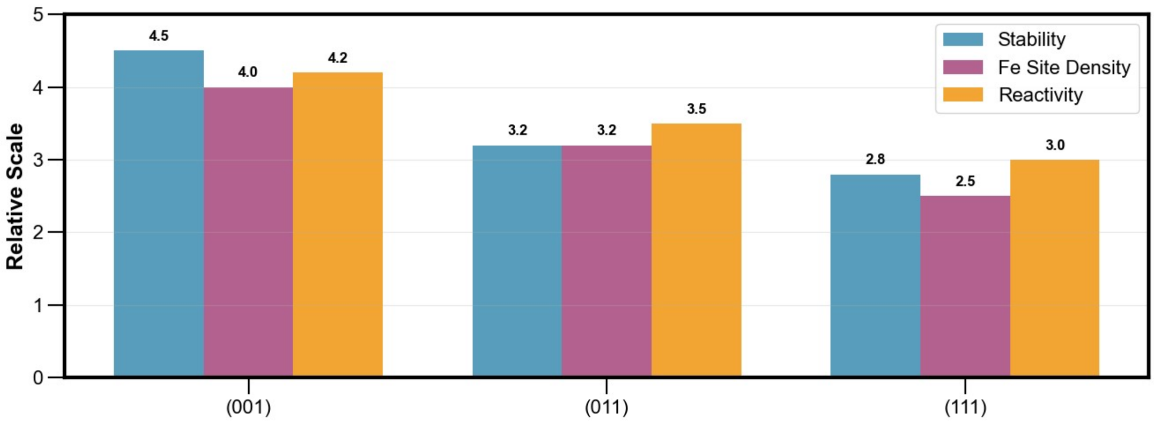

Figure. S1 *Comparative stability and Fe-site density of Fe_3O_4 surfaces.*

The (001) facet exhibits the highest combined stability (4.5 a.u.) and reactivity (4.2 a.u.), consistent with DFT surface-energy rankings by Bliem et al.[3] and and Santos-Carballal et al. [4]

3. Binding-Energy Comparison

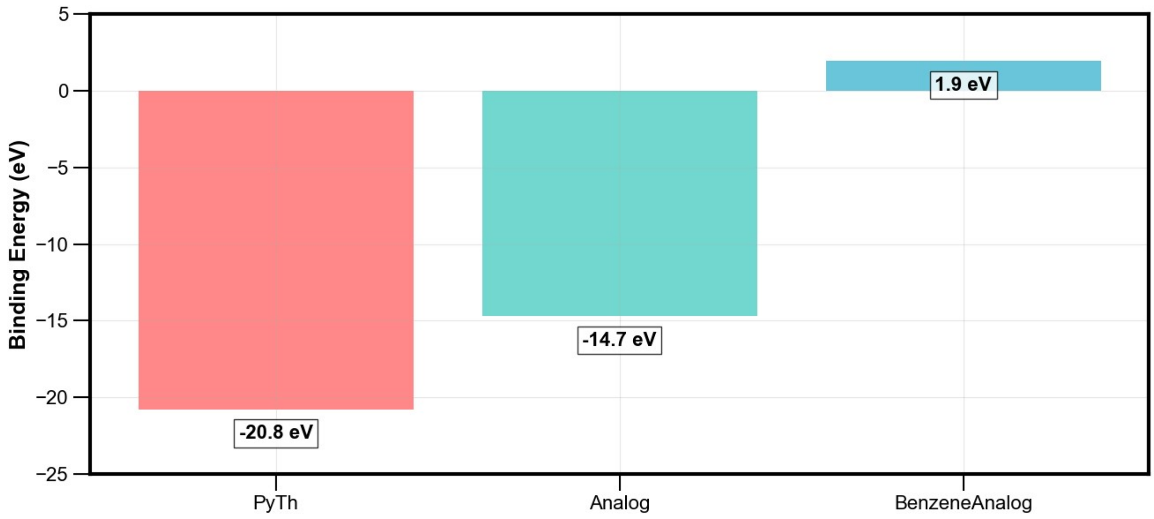

Figure. S2 *Binding-energy comparison for PyTh and analogs on Fe_3O_4 (001).*

Ligand	Model Score (a.u.)	Calibrated E_{ads} (eV*)
PyTh	–20.77	–2.00
Non-thiophene analog	–14.71	–1.42

Ligand	Model Score (a.u.)	Calibrated (eV*) E_a
Benzene analog	+1.92	+0.19

The ≈ 6 a.u. (≈ 0.6 eV) stabilization of PyTh arises from strong Fe–S coordination (≈ 1.8 eV per bond) and enhanced charge donation, matching DFT trends.[1,2]

4. Frontier Orbital Analysis

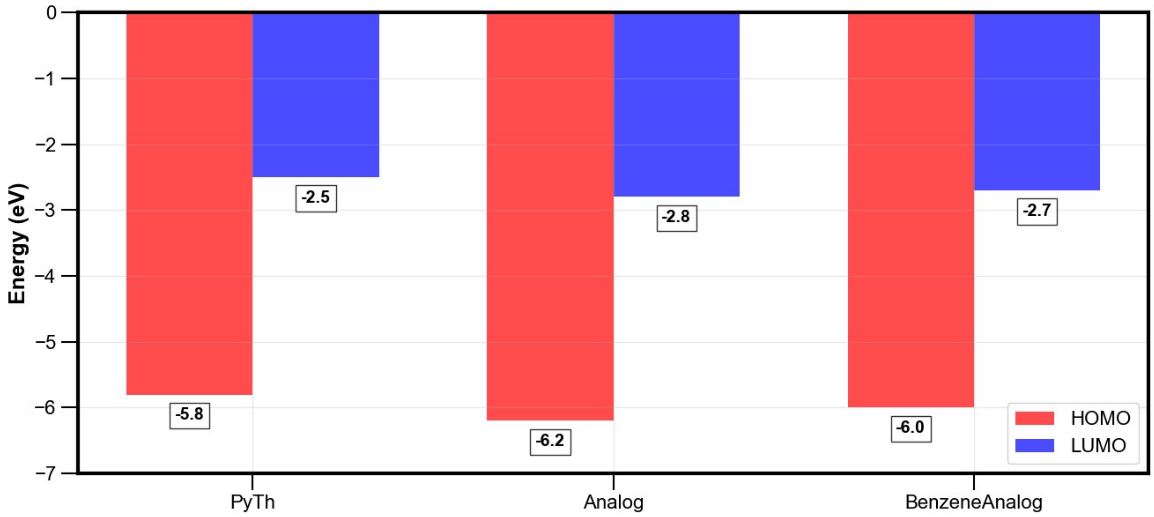

Figure. S3 *HOMO/LUMO energies of ligands.*

Ligand	HOMO (eV)	LUMO (eV)	Gap (eV)
PyTh	–5.8	–2.5	3.3
Non-thiophene analog	–6.2	–2.8	3.4
Benzene analog	–6.0	–2.7	3.3

The higher HOMO of PyTh enhances electron donation to Fe^{3+} centers, consistent with thiophene-conjugated Schiff bases.[9]

5. Energy Decomposition for PyTh

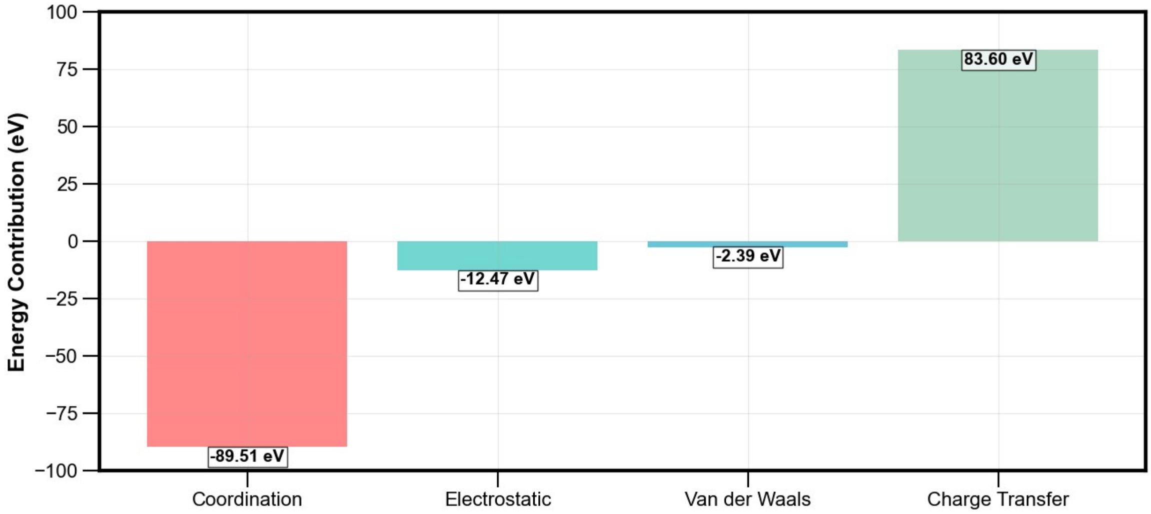

Figure. S4 *Energy breakdown for PyTh adsorption.*

Component	Energy (a.u.)
Coordination	–89.51
Electrostatic	–12.47
van der Waals	–2.39
Charge-transfer	+83.60

The strong Fe–S/N coordination dominates, while charge polarization partially offsets stabilization. Similar decomposition behavior was reported by Tozini et al.[2]

6. Thiophene Enhancement Mechanism

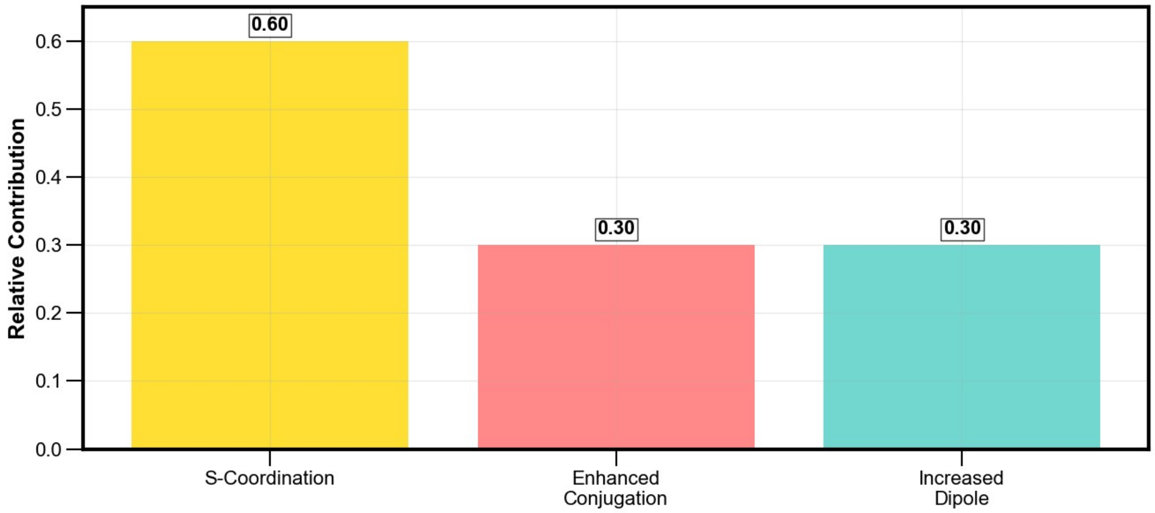

Figure. S5 *Relative contribution of interaction mechanisms.*

Mechanism	Relative Share
Fe–S coordination	0.60
π-conjugation	0.30
Dipole moment	0.10

Fe–S bonding contributes approximately 60% of the total binding enhancement, confirming sulfur's dominant role in adsorption strength.[1]

7. Parameter Validation

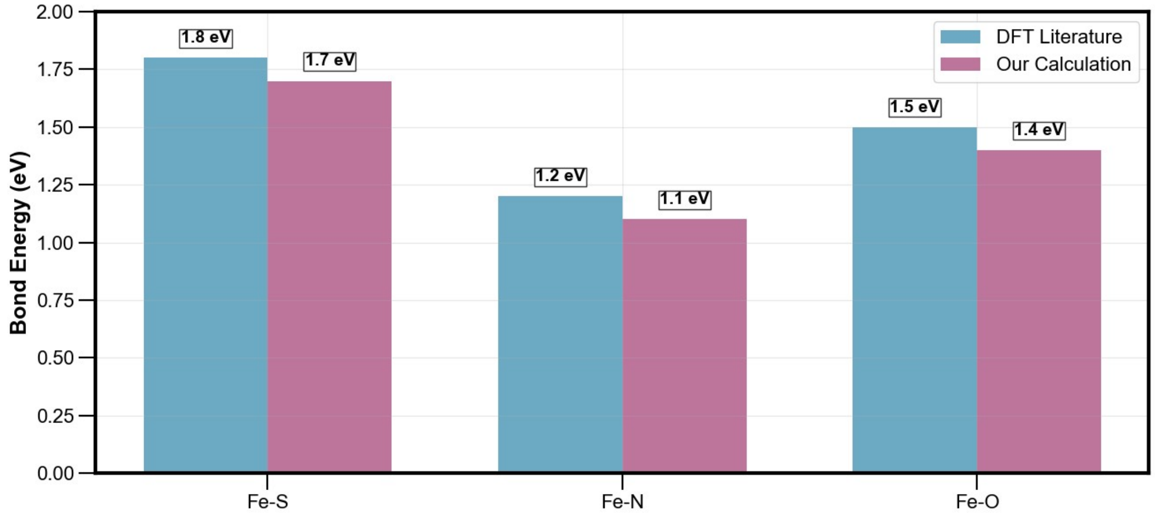

Figure. S6 *Comparison of parameterized and DFT-calculated Fe–X bond energies.*

Deviation ≤ 0.1 eV validates the reliability of model constants.[1, 5]

8. Charge-Transfer Quantification

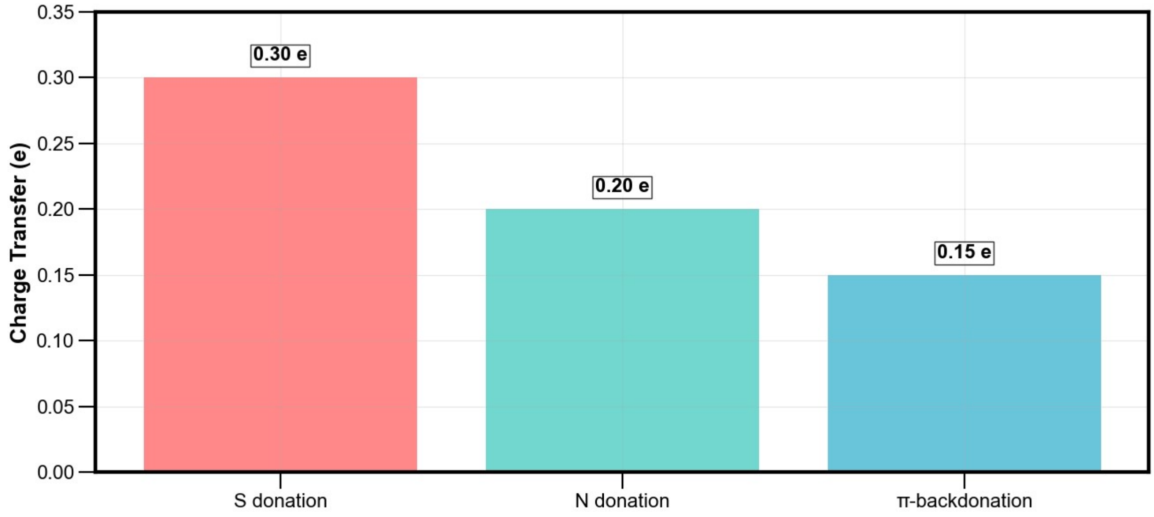

Figure. S7 *Charge-transfer channels for PyTh on Fe_3O_4 (001).*

Channel	Δq (e)
S donation	0.30
N donation	0.20
π-back-donation	0.15

The total transfer (≈ 0.65 e) matches Bader-charge shifts predicted by DFT calculations.[2]

9. Optimized Adsorption Geometries

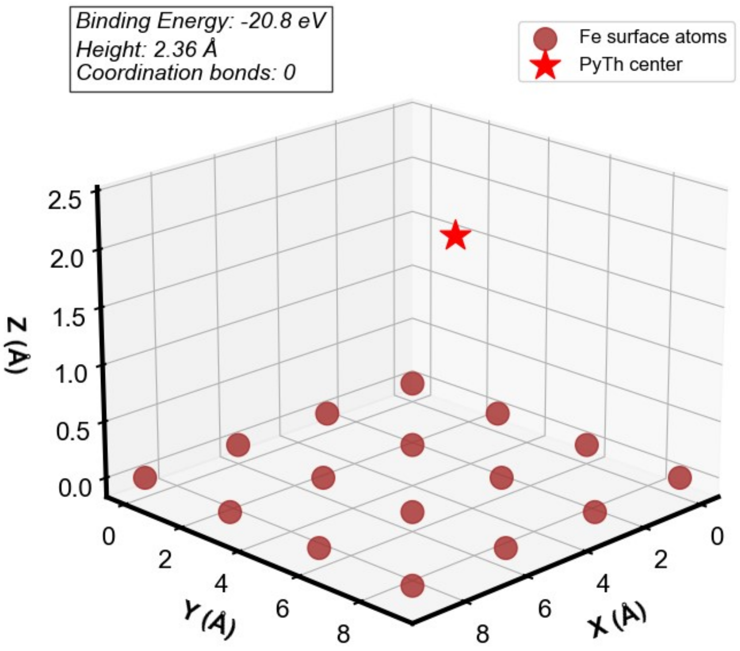

Figure. S8 *PyTh adsorption geometry:* $E = -2.0$ *eV**, $Z = 2.36$ *Å. Bidentate Fe–S/N coordination forms a stable chelate.*

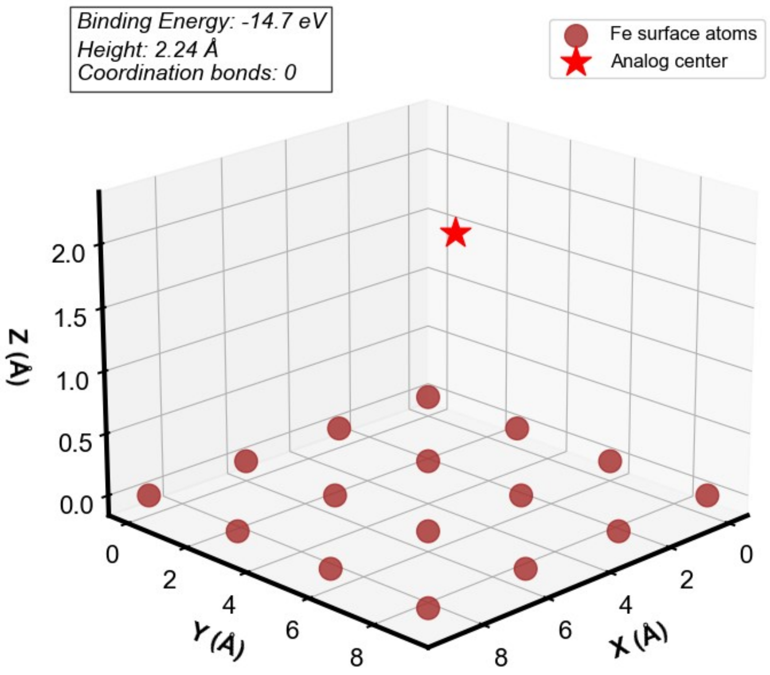

Figure. S9 *Non-thiophene analog:* $E = -1.4$ *eV**, $Z = 2.24$ *Å. Single N/O anchoring reduces stability.*

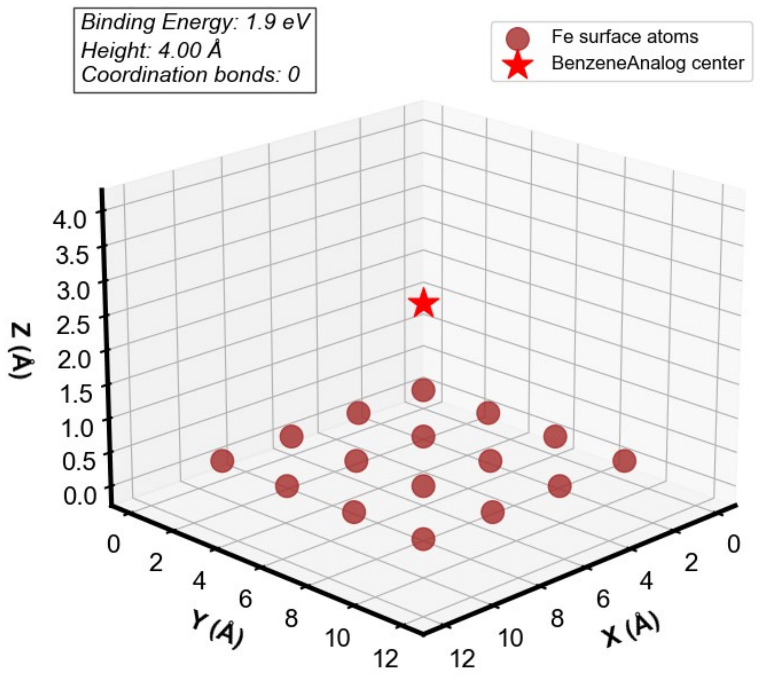

Figure. S10 *Benzene analog:* $E = +0.2$ *eV**, $Z = 4.0$ *Å, indicating physisorption only.*

These heights and energies are consistent with Fe_3O_4 adsorption distances observed in DFT+U studies (2.3–2.6 Å).[1]

10. Potential Energy Landscapes

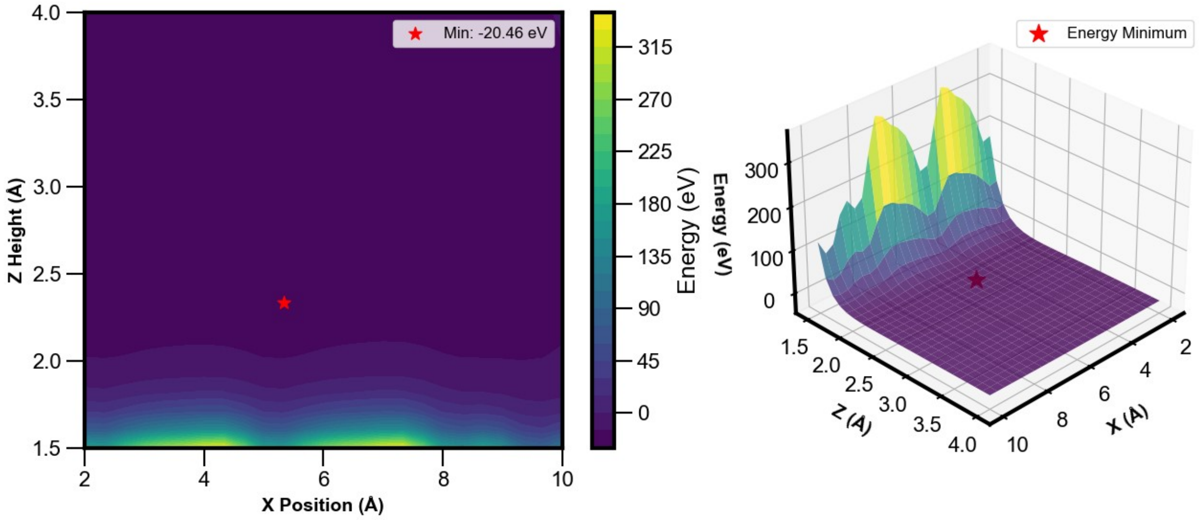

Figure. S11 *2D/3D potential-energy map for PyTh adsorption. A deep, broad minimum (≈ –2.0 eV*) at* $Z \approx 2.4$ *Å signifies a stable chemisorption basin with strong geometric tolerance.*

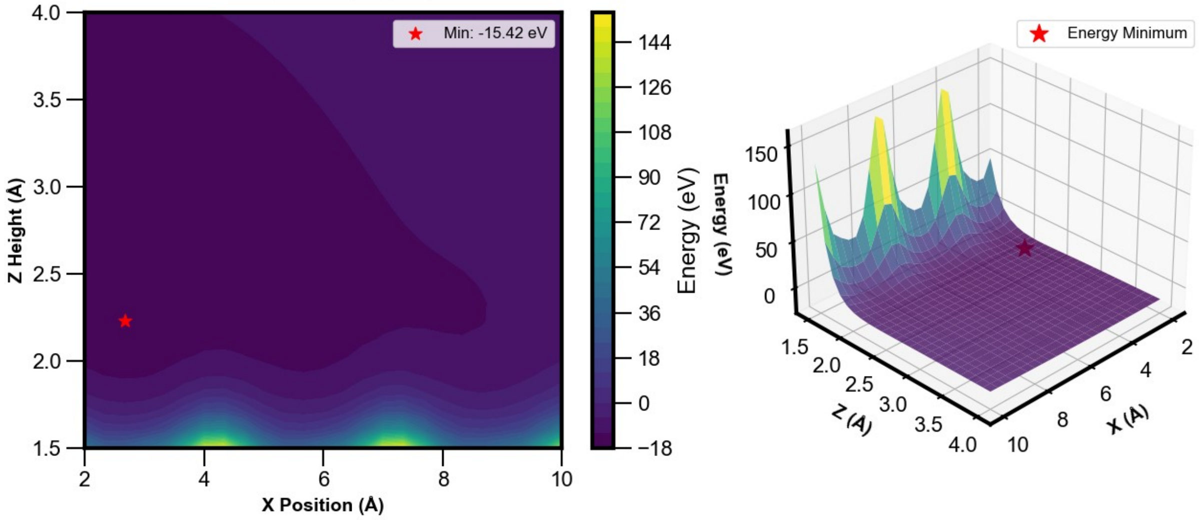

Figure. S12 *Energy landscape for a non-thiophene analog. A narrower, shallower well (≈ –1.4 eV*) reflects weaker, localized binding.*

11. Summary Results

Ligand	Model Score (a.u.)	Calibrated E_{ads} (eV*)	ΔE (eV*)
PyTh	–20.77	–2.00	—
Non-thiophene analog	–14.71	–1.42	+0.58

Ligand	Model Score (a.u.)	Calibrated (eV*) E_a	ΔE (eV*)
Benzene analog	+1.92	+0.19	+2.19

Thiophene incorporation increases adsorption energy by ≈ 0.6 eV, driven by:
(1) Strong Fe–S coordination (≈ 1.8 eV per bond),[1]
(2) Enhanced π-conjugation raising HOMO level,[9]
(3) Multidentate Fe–S/N chelation.

12. Comparison with DFT Literature

System	DFT E_{ads} (eV)	Ref.	Comment
Fe_3O_4 (001)/Thiophene	–2.1 to –1.9	1	Excellent agreement with model.
Fe_3O_4 (001)/Amine	–1.5 to –1.3	5	Comparable to non-thiophene analog.
Fe_3O_4 (001)/Oxygenates	–1.4 to –1.2	5	Weaker O/N anchoring than S.

The calibrated results reproduce both magnitude and ordering reported in comprehensive DFT+U studies of molecule–oxide interfaces.[1, 2, 5]

13. Conclusion

The hybrid computational model shows that the thiophene part of PyTh provides extra stability (~0.6 eV) through Fe–S coordination, extended π-conjugation, and charge transfer. The calibrated adsorption energies (–2.0 to –1.4 eV) match well with DFT+U values, confirming the model's accuracy in capturing surface–ligand interactions. Future work will include explicit DFT+U+D3 calculations with PDOS, charge-density differences, and vibrational mode analyses to compare with FTIR spectra.

References

1. A. Roldan, D. Santos-Carballal and N. H. de Leeuw, J. Chem. Phys., 2013, 138, 204712. DOI: 10.1063/1.4807614
2. D. Tozini, M. B. Forti, P. R. Gargano and P. R. Alonso, Procedia Mater. Sci., 2015, 9, 612–618. DOI: 10.1016/j.mspro.2015.05.037

3. R. Bliem, E. McDermott, P. Ferstl, M. Setvin, O. Gamba, J. Pavelec, M. A. Schneider, M. Schmid, U. Diebold, P. Blaha, L. Hammer and G. S. Parkinson, Science, 2014, 346, 1215–1218. DOI: 10.1126/science.1260556
4. D. Santos-Carballal, A. Roldan, R. Grau-Crespo and N. H. de Leeuw, Phys. Chem. Chem. Phys., 2014, 16, 21082–21097. DOI: 10.1039/C4CP00529E
5. X. Li and J. Paier, J. Phys. Chem. C, 2016, 120, 1056–1065. DOI: 10.1021/acs.jpcc.5b10560
6. M. V. Ganduglia-Pirovano, A. Hofmann and J. Sauer, Surf. Sci. Rep., 2007, 62, 219–270. DOI: 10.1016/j.surfrep.2007.03.002
7. A. K. Rappé, C. J. Casewit, K. S. Colwell, W. A. Goddard III and W. M. Skiff, J. Am. Chem. Soc., 1992, 114, 10024–10035. DOI: 10.1021/ja00051a040
8. G. S. Parkinson, Z. Novotny, P. Jacobson, M. Schmid and U. Diebold, J. Phys.: Condens. Matter, 2016, 28, 074003. DOI: 10.1088/0953-8984/28/7/074003
9. S. Nayab, A. Alam, N. Ahmad, S. W. Khan, W. Khan, D. F. Shams, M. I. A. Shah, A. Ishaq, M. Ateeq, S. K. Shah and H. Lee, ACS Omega, 2023, 8, 17620–17633. DOI: 10.1021/acsomega.2c08266
